# Osteopontin regulates biomimetic calcium phosphate crystallization from disordered mineral layers covering apatite crystallites

**DOI:** 10.1038/s41598-020-72786-x

**Published:** 2020-09-24

**Authors:** Taly Iline-Vul, Raju Nanda, Borja Mateos, Shani Hazan, Irina Matlahov, Ilana Perelshtein, Keren Keinan-Adamsky, Gerhard Althoff-Ospelt, Robert Konrat, Gil Goobes

**Affiliations:** 1 Department of Chemistry, Bar Ilan University, 5290002 Ramat Gan, Israel; 2 Max F. Perutz Laboratories, Department of Computational and Structural Biology, University of Vienna, 1030 Vienna, Austria; 3 Solid-State NMR Application, Bruker Biospin GmbH, Rheinstetten, Germany

**Keywords:** Solid-state NMR, Biomineralization

## Abstract

Details of apatite formation and development in bone below the nanometer scale remain enigmatic. Regulation of mineralization was shown to be governed by the activity of non-collagenous proteins with many bone diseases stemming from improper activity of these proteins. Apatite crystal growth inhibition or enhancement is thought to involve direct interaction of these proteins with exposed faces of apatite crystals. However, experimental evidence of the molecular binding events that occur and that allow these proteins to exert their functions are lacking. Moreover, recent high-resolution measurements of apatite crystallites in bone have shown that individual crystallites are covered by a persistent layer of amorphous calcium phosphate. It is therefore unclear whether non-collagenous proteins can interact with the faces of the mineral crystallites directly and what are the consequences of the presence of a disordered mineral layer to their functionality. In this work, the regulatory effect of recombinant osteopontin on biomimetic apatite is shown to produce platelet-shaped apatite crystallites with disordered layers coating them. The protein is also shown to regulate the content and properties of the disordered mineral phase (and sublayers within it). Through solid-state NMR atomic carbon-phosphorous distance measurements, the protein is shown to be located in the disordered phases, reaching out to interact with the surfaces of the crystals only through very few sidechains. These observations suggest that non-phosphorylated osteopontin acts as regulator of the coating mineral layers and exerts its effect on apatite crystal growth processes mostly from afar with a limited number of contact points with the crystal.

## Introduction

Calcium phosphate mineralization processes are key to constructing well-organized mechanically viable structures in tissues like bone^1,2^, tooth enamel, dentin and cementum. One of the more complex mineralized tissues is no doubt bone. Bone can take different densities and compositions of minerals depending on the mechanical task it is destined to perform and the maturation stage of the tissue. Assembled collagen fibers adopt various ordered and disordered architectures depending on their location in the bone matrix and the type of bone^3–7^, providing with the scaffold for calcium phosphate to precipitate and transform into apatite^8^. A substantial part of bone mineralization occurs through apatite precipitation inside and around collagen fibrils^9,10^. Biomimetic mineralization of collagen involves precipitation of the precursor inorganic ions in the presence of polyaspartate which leads to formation of apatite^11–13^. This polyelectrolyte is used as model molecule for the acidic non-collagenous proteins (NCPs) in bone, known to regulate mineral formation^14–16^. The interplay of collagen, NCPs and other constituents such as water, citrate ions, glycosaminoglycans (GAG) in forming tight interfaces that provide bone with its exquisite mechanical properties is the subject of extensive ongoing investigations^17^ and few controversies^18,19^. Recent work has shown that collagen functions in synergy with polyaspartate as an inhibitor of mineral nucleation to actively control mineralization. In another work, GAGs were shown to interact directly with the mineral phase in bones^20^. Other studies suggested that collagen is able to initiate the mineralization process without active involvement of other biomolecules^9^, and emphasized the role of water and citrate ions in orienting and spacing out apatite crystal platelets^21^.

## Biological activity of non-colagenous proteins

The specific functions of many NCPs remain elusive and more so the way these activities fit into an integrative picture of inorganic material design in bone. Their unusual primary structure, with segments that are non-homologous with other known proteins, may indicate diverse functions, some of which still uncovered. Many of the NCPs were shown to be directly involved in control of hydroxyapatite (HAP) crystallization^22^; some can inhibit crystal nucleation or growth while others promote and speed up the process^23^. In addition, non-collagenous proteins impart important functions in cell signaling and recruitment and in maintaining ion homeostasis. Their regulatory capabilities over bone mineral and bone cell activities, indicates that they may be influencing bone mineralization in more than one pathway.

As different NCPs may perform complementary functions in mineralization, there is a need to compare the activity of a range of NCPs in bone-mimetic and enamel-mimetic mineralization^24^ in vitro and to infer in atomic detail the changes they may impose on the different mineral phases that are precipitated. Such studies are also extremely important to deciphering mineralization in other organisms^25^. Recently, we have studied the regulatory effect on the in vitro crystallization of bone-mimetic apatite of two bone-associated polypeptides. Osteocalcin, unmodified posttranslationally^26^ (umOC) and ON29, a peptide from osteonectin’s mineral binding domain^27^ exhibited markedly different effects on the process of crystallization. Apart from their disparate effects on the morphology and crystallinity of apatite, we could show that the two biomolecules also intervened with the thickness and composition of the amorphous mineral layer, coating the apatite crystallites; ON29 induced thickening of this layer, while OC promoted the reverse. These studies revealed a new partially ordered mineral phase interconnecting the crystalline and amorphous phases we termed the “inter-phase”. The existence of such intermediate mineral phase alleviates the stringent transition from a completely disordered hydrated phase to a highly ordered core crystalline phase. ON29 and umOC co-precipitated with the mineral were capable of changing the content of HPO_4_^2−^ ions and water molecules in the hydrated layer as well^26–29^. These findings open new questions regarding the capabilities possessed by non-collagenous proteins, for regulating and modifying the mineral phases via organic–inorganic interactions.

## Osteopontin biological activity

One of the extensively studied NCPs is osteopontin (OPN), named for its role as a bridge between cells and minerals. OPN is a bone matrix glycoprotein found in bone and teeth. The protein is a large intrinsically disordered protein (IDP) highly enriched in acidic sidechains; a quarter of its sequence contains acidic aspartic or glutamic residues. The protein is also extensively phosphorylated post-translationally as part of its functional regulation^30–32^. The protein is classified as a matricellular cytokine^33,34^ serving as an important factor in bone remodeling and in bone formation under mechanical stress^35^. However, the exact function of OPN in the mineralization process is still unclear as its activity in higher tasks such as cell signaling were not completely worked out yet^36,37^. OPN and other NCPs are members of the SIBLING family (small integrin binding ligand N-linked glycoproteins) which contain the ASARM motif (the acidic serine-and aspartate-rich)^30^. Phosphorylated ASARM peptides are known to bind hydroxyapatite crystals and inhibit extracellular matrix mineralization^38^. This inhibition is regulated by the protease PHEX^39,40^ which can cleave OPN to fragments so small that they retain no activity in mineral regulation anymore.

The effect of the phosphorylation of OPN peptides on calcium phosphate mineral formation, growth, and mineralization inhibition was examined by several groups^17,41–43^. The protein binds divalent cations with high affinity in solution^44^ and may act as an inhibitor and as a nucleator of apatite, depending on its concentration, the state of phosphorylation and the abundance of acidic amino acids along its sequence^23,32,45^. A phosphorylated synthetic short peptide derived from OPN was shown to inhibit HAP growth more effectively than the very acidic DDDDDD peptide^46^. The pH used in these measurements was set to physiological value (pH 7.4) at which HAP solubility was higher thus promoting formation of other calcium-phosphate phases. A recent work has challenged the relation between the phosphorylation state in OPN and its calcium binding ability^37,44^.

## Structural features of osteopontin

Early studies using NMR showed that OPN, along with other SIBLING proteins, has a flexible structure in solution^47^. At about the same time, other NCPs were suggested to be intrinsically disordered in solution^48,49^, leading to a new paradigm relating high conformational flexibility to regulation capabilities of mineral crystal growth through motions^36^. Recent NMR analysis confirmed that OPN is intrinsically disordered in solution^50–52^. Using ^13^C secondary shifts, local secondary structure elements were identified having reduced conformational flexibility. The correlation between dynamic heterogeneity along the protein backbone and deviations from random coil chemical shifts provided information of local structure motifs with motion-restricted residues suggesting stable local conformations. Analysis of the protein's meta-structure provided quantitative information about regional compactness and topology in the protein^50^. At the same time, the structures of the charged domains, noted as potential binding motifs to the mineral phase, are still challenging to determine experimentally^47^.

## Functions of osteopontin in bone mechanics

The contribution of NCPs to mechanical viability of bone, acting as key interfacial constituents in organic–inorganic composite structure, was lately investigated^53^. Genetic knockout mice models were used in order to explore the role of OPN and osteocalcin (OC) proteins jointly and separately in bone toughness and in tissue response to micro cracking^54^. Bone fracture was shown to involve the formation of dilatation bands as a result of OC-OPN complex interaction. In that study, OPN was docked to extrafibrillar mineral via the linker protein OC and participated in greater energy dissipation upon mechanical load. The toughness of bone was shown to decrease dramatically in mice lacking OC, OPN or both. OPN deficiency alone led to a 30% decrease in mice femur fracture toughness and to a larger variability in calcium concentration in the bone^53^. The atomic details of the organic–inorganic interfaces in these genetic knockout bones were compared to wild-type bones by solid-state NMR^55^, showing some modulation in lysine/arginine residues and GAG/citrate molecules content at the interfaces. Evidently, fundamental analysis of the organic-mineral interface in the context of the entire tissue is still challenging and more so will be to identify subtle differences taking place during early mineralization event with OPN, though recent in vitro work reported changes to mineral ion clustering caused by the protein during crystallization^56^.

## Mineral phases and osteopontin-mineral interface

In the present study, the influence of a recombinant OPN comprising residues 45 to 264 of the quail *Coturnix japonica* on bone-like mineral formation was examined. Bone-mimetic apatite was crystallized in the presence of quail osteopontin (qOPN) under physiological temperature and using fixed pH conditions. The effect of the biomolecule on composition, phase, and morphology of the forming mineral were analyzed by solid-state NMR spectroscopy, powder X-ray diffraction (XRD), electron microscopy (EM), Brunauer-Emmet-Teller (BET) gas adsorption isotherms, inductively coupled plasma (ICP) and elemental analysis (EA).

The protein is shown to modulate the properties of the disordered mineral layers coating the core apatite crystallite. Relayed ^13^C–^31^P magnetization transfer NMR measurements indicate that the protein is closer to phosphates in the disordered layers than to phosphates in the core apatite phase, consistent with its prominent effect on the properties of this phase mostly. Direct proximity measurements from protein carbons to mineral phosphates further corroborate this, by indicating only few contact points between the protein and the apatite crystal surface.

## Results and discussion

### Electron microscopy, diffraction and surface area measurements

**Figure 1:**
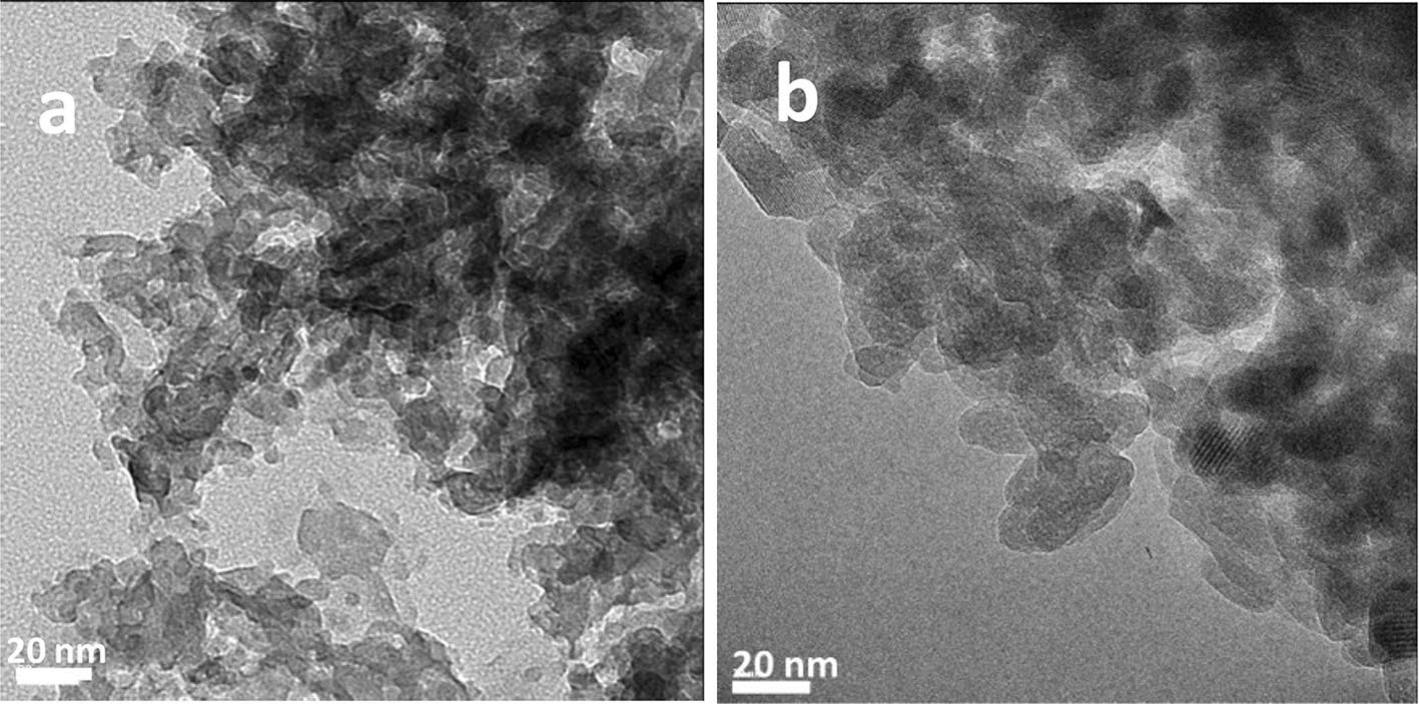
Platelet-shaped crystallites observed in the transmission electron micrographs of (**a**) HAP and (**b**) HAP·qOPN.

HRTEM images of HAP (a) and HAP·qOPN (b) are shown in Fig. 1. These images show that the morphology of individual crystallites is plate-like in both samples, reminiscent of the apatite crystallites in bone. The average plate size is 10–20 nm for apatite and 10–15 nm for apatite formed with qOPN. Electron diffraction analyses of crystallites from selected area of the two samples are shown in Figs. S1–S3, confirming that the crystallites are comprised of hexagonal hydroxyapatite.

**Figure 2:**
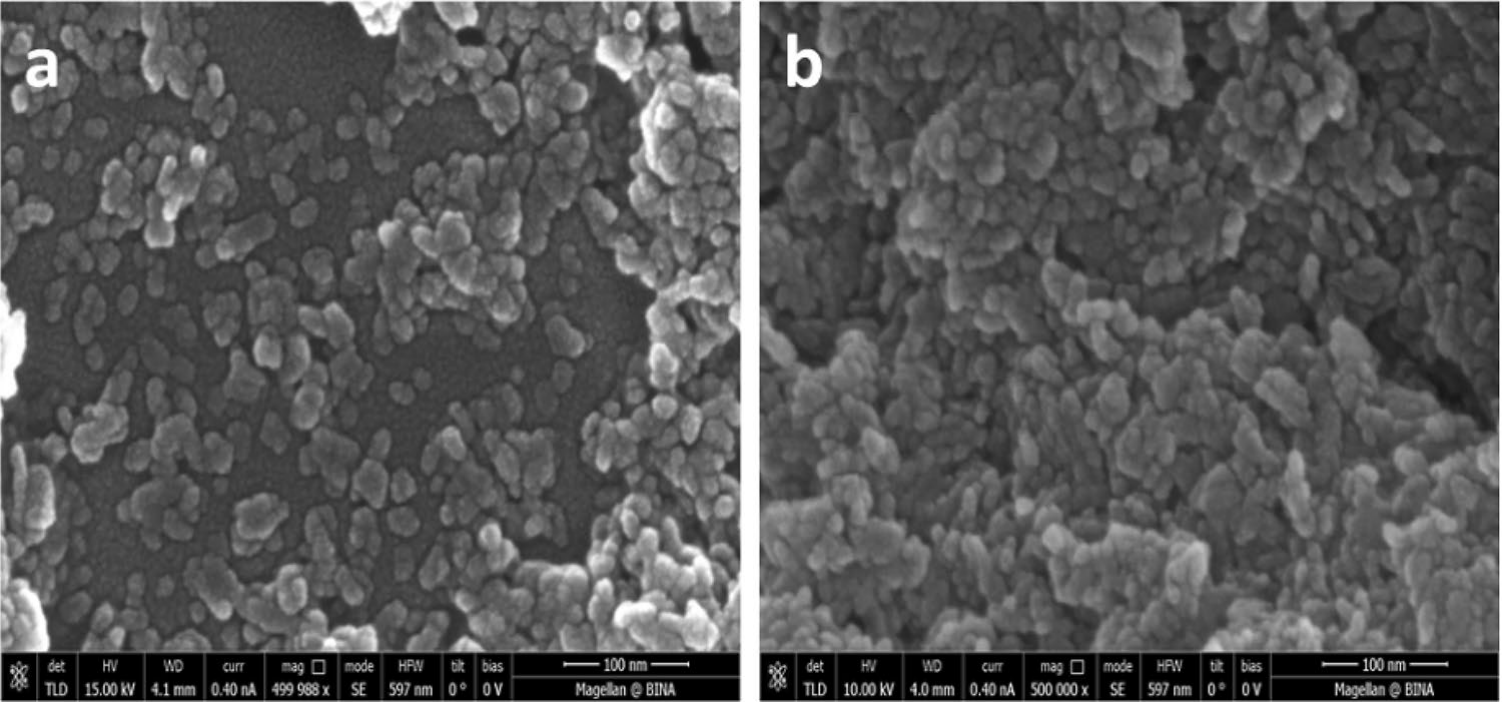
Agglutination of apatite particles observed from scanning electron micrographs of (**a**) HAP and (**b**) HAP·qOPN.

Powder X-ray diffractograms of HAP (red) and HAP·qOPN (blue) are shown in Fig. S4. The reflection pattern of HAP·qOPN is showing slightly broader lines and lower S/N ratio than HAP suggesting smaller crystallites were formed with the protein. Scherer analysis of the 25.9° reflection width, accordingly, gave a crystallite size of 24(± 2) nm for HAP and 20(± 2) nm for HAP·qOPN. The crystallites size in the electron micrographs for both samples are in reasonable agreement with the Scherer analysis of the XRD results. The Scherer equation gives a crude upper boundary value of the crystallites size and is affected by factors such as non-uniform lattice distortions, grain surface relaxation, dislocations and instrumental peak profile. HRSEM images of HAP and HAP·qOPN are shown in Fig. 2. The crystallites appear denser in the HAP·qOPN image as they were more challenging to disperse in the presence of the protein. Brunauer-Emmet-Teller isotherms were used to record the specific surface area (SSA), of HAP 185(± 19) m^2^/g and HAP·qOPN 99(± 10) m^2^/g. These values indicate lower surface is available for nitrogen adsorption in the mineral prepared with qOPN.

### Elemental and thermogravimetric analysis

Inductively coupled plasma (ICP) analysis was performed to determine the bulk calcium to phosphate ratio. The measurements were carried out in several repeats and verified against commercial nano HAP from Sigma-Aldrich, which gave a value of 1.70. The Ca/P ratio measured for HAP without qOPN protein was 1.66 and the ratio for HAP·qOPN was 1.78 (the stoichiometric value for hydroxyapatite is 1.67).

Elemental analysis results of the two samples are summarized in Table 1. HAP contains 1.1 wt% of carbon precipitating with mineral ions, indicating that under the conditions used some carbonates are incorporated into the apatite during synthesis from residual CO_2_ gas dissolved in the precipitation solution. Elemental analysis of HAP in the presence of the protein indicates that a substantial amount of the protein precipitates with the mineral. The C/N ratio in HAP·qOPN is 3.52 (Table 1) which is higher than the calculated ratio for qOPN, 3.28 based on its sequence. The difference is attributed to incorporation of carbonate ions into the mineral during its formation, similarly to the HAP sample.

**Table 1:** Elemental analysis of HAP and HAP·qOPN (values reported in wt%).

	Nitrogen	Carbon	Hydrogen	Oxygen
HAP	–	1.13	1.39	9.71
HAP·qOPN	2.21	7.79	1.81	14.24

TGA analysis of HAP·qOPN (Fig. S5) shows that the protein constitutes 15% weight of the complex (~ 0.25 mol%). Comparison of the normalized weight loss with temperature after accounting for slight differences in water evaporation in HAP·qOPN and in HAP is shown in Fig. S5. Combustion of the organic material is observed from 250 °C to 550 °C followed by another weight decrease of ~ 1.6% between 750 °C and 850 °C. The sigmoidal shape of the weight loss curve during pyrolysis of the qOPN may suggest a cooperative process, putatively of desorption, that precedes combustion of the molecules. Differential thermal analysis, also shown in Fig. S5, indicates that pyrolysis of qOPN is maximal at a temperature of 350 °C.

### Solid state NMR analysis of the crystalline and disordered mineral phases

#### ^31^P direct polarization and ^1^H–^31^P cross polarization

^31^P direct polarization (DP) and cross polarization (CP) MAS spectra of the two samples are shown in Fig. S6. The phosphate lines of HAP (shown in blue) are slightly narrower than that of HAP·qOPN (shown in red) in both experiments, indicating a larger dispersion of the phosphate chemical shifts in the mineral prepared with the protein. As was shown before for HAP·ON29 and HAP·umOC, each of these phosphate lines, in fact, comprises several phosphate species, from the bulk of the crystalline material and from disordered mineral layers that cover the apatite crystals. This is further corroborated using 2D ^1^H–^31^P HETCOR measurements which separate out phosphates based on protons in their vicinity. ^31^P chemical shift anisotropy of 14.8 (± 0.2) kHz and asymmetry of 1.0 (± 0.1) were measured for both materials from analysis of the sideband intensities in ^31^P CP spectra recorded at slow spinning rates using the DMFIT program^57^ suggesting that the immediate environment of the PO_4_^3−^ ions is not affected by the preparation with qOPN.

**Figure 3:**
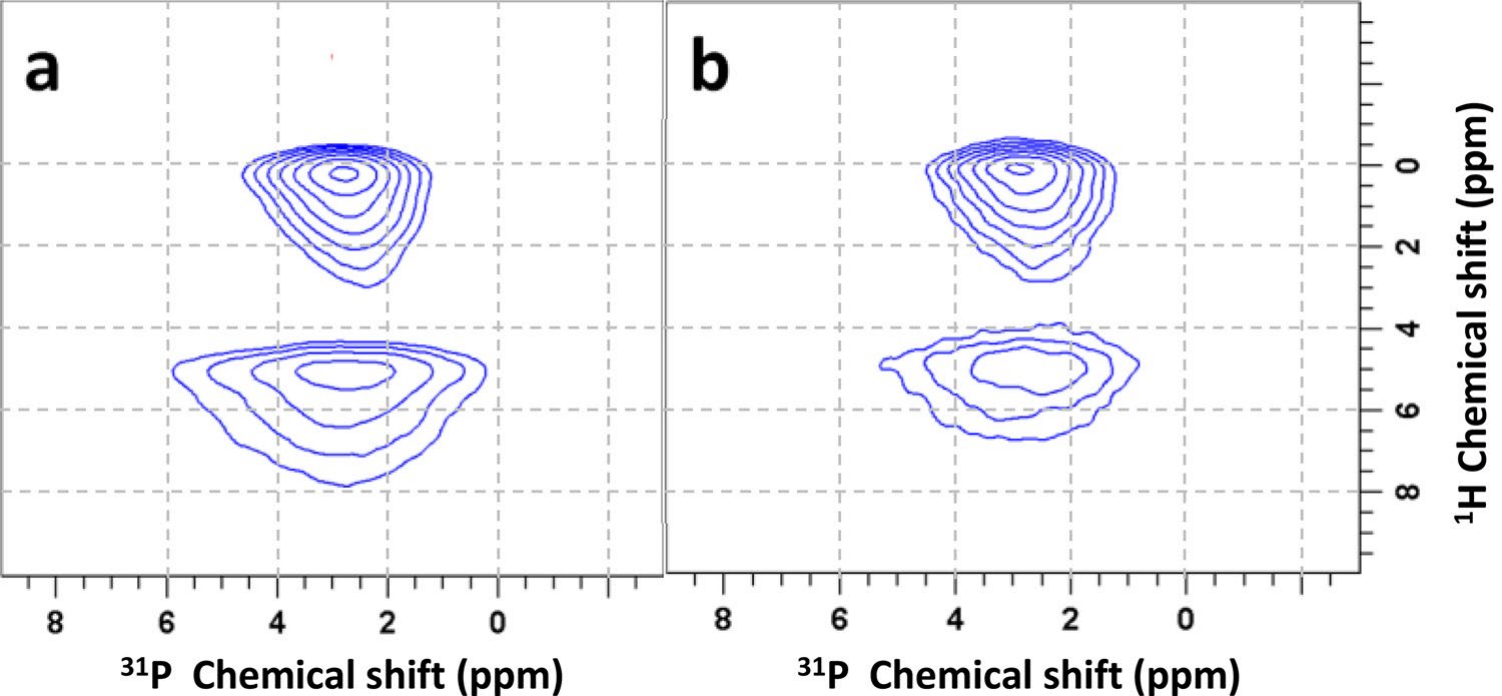
2D ^1^H–^31^P HETCOR spectra of HAP (**a**) and HAP·qOPN (**b**) recorded using the PMLG pulse scheme in t_1_ for ^1^H homonuclear decoupling and a CP contact time of 0.8 ms.

To examine the individual magnetization transfers from different protons to phosphate species in HAP and HAP·qOPN, 2D ^1^H–^31^P HETCOR experiments, utilizing ^1^H–^1^H decoupling during ^1^H evolution in t_1_, were performed. The use of PMLG decoupling with sample spinning at a rate of 10 kHz is sufficient to average out ^1^H–^1^H dipolar couplings between ^1^H-containing mineral ions (OH^−^ and HPO_4_^2−^) and mobile water molecules but not to remove the couplings between the protons on qOPN and highly immobile water, therefore signals from protein protons near phosphate groups are not visible in the spectra. Two cross peaks are observed in the spectra of the two material, shown in Fig. 3. A peak connecting phosphates with a ^1^H resonance of OH^−^ at 0.2 ppm representing mostly the bulk of apatite crystals and another peak connecting phosphates with ^1^H resonances of H_2_O at 5.1 ppm representing the hydrated layer of the mineral that covers each crystallite. The hydrated layer cross peak is broader along the ^31^P axis indicating that the phosphates in this layer are disordered, as shown before for cortical bone^10^ and synthetic apatite preparations with^27^ and without^58^ bone proteins. The peaks are broad along the ^1^H chemical shift axis due to heterogeneity in the sample which leads to chemical shift dispersion. For skyline projections along the ^1^H and ^31^P chemical shift axes in these 2D spectra see Fig. S7.

**Figure 4:**
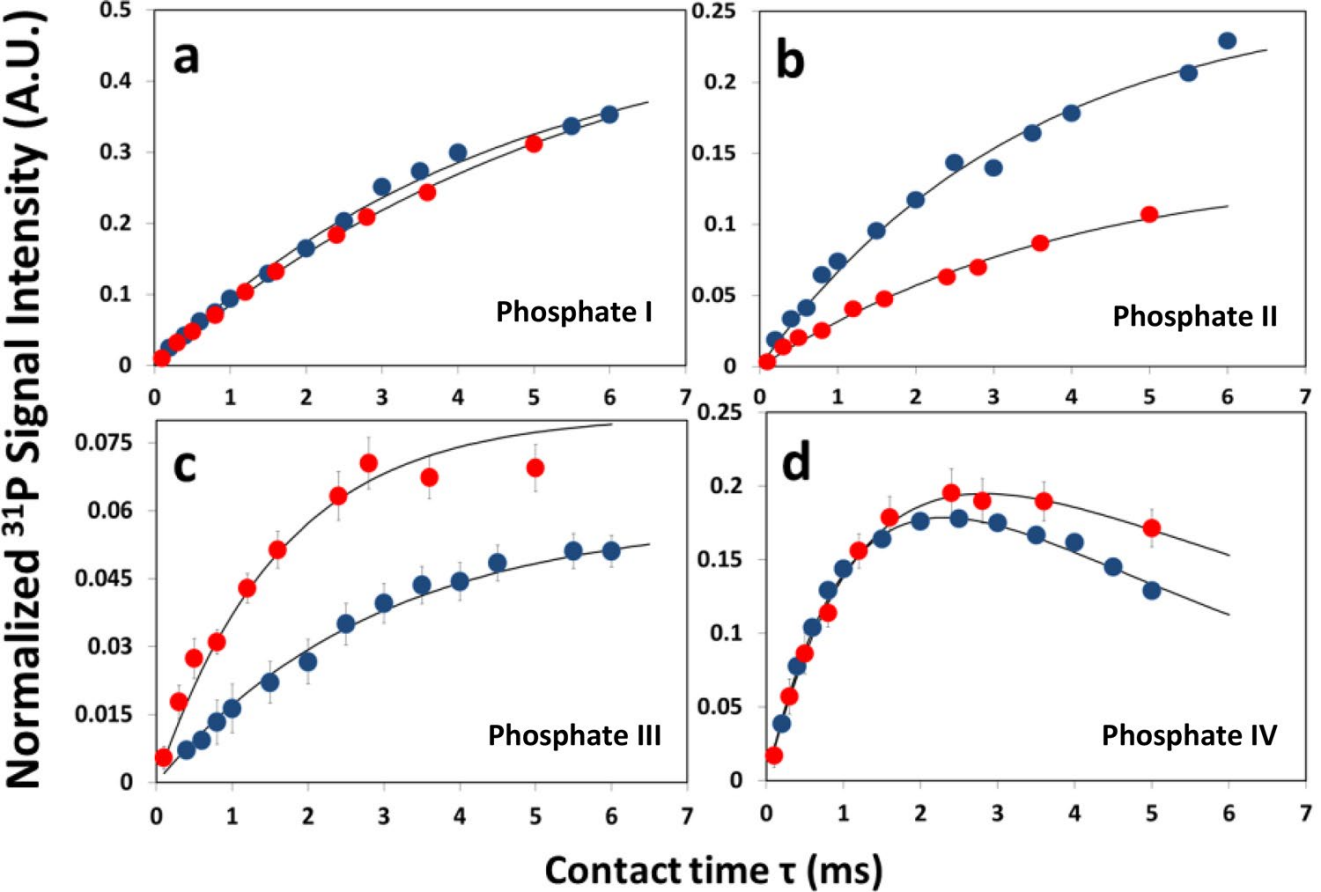
^31^P signal intensity buildup measured by varying the contact time in ^1^H–^31^P HETCOR measurements of HAP (red) and HAP·qOPN (blue). Graphs a and b show hydroxyl transfer to crystalline and partially disordered phosphates, respectively. Graphs c and d show water transfer to partially ordered and to disordered phosphates, respectively. In graphs (**a**) and (**b**) and partially in (**d**), calculated errors are within the size of symbols. HAP data shown were also reported before.

Interestingly, the NMR properties of the phosphates in this layer are sensitive to the protein used in the mineral synthesis. Here, for example, the disordered phosphate peak in HAP·qOPN is narrower than in HAP, indicating that the protein acts to decrease the degree of disorder in the surface layer, as was observed for umOC^26^. The reverse effect was observed for ON29, whereby the peptide induced greater disorder in this layer^27^. The surface layer cross peak in HAP·qOPN is also less intense, however, since quantitative intensities in CP experiments are generally challenging, it can be suggested that the relative amount of amorphous mineral layer is smaller in the presence of the protein, under the assumption of similar CP dynamics in two very similar preparations.

The cross peaks in the HETCOR spectra of the two materials comprise more than one phosphate species as evidenced from their asymmetric shape in a projection onto the ^31^P axis. One-dimensional ^31^P projections of the cross-peaks were, therefore, taken by slicing the HETCOR spectra in Fig. 3 horizontally at 0.2 ppm and at 5.1 ppm. The resultant four projections were deconvolved using a two-line analysis in the DMFIT program. Example fittings for the OH^−^ projections are shown in Fig. S8 for the two materials. The four phosphate species obtained for HAP·qOPN by fitting the H_2_O and OH^−^ projections are shown in Fig. S9. Similarly, four phosphates are derived from analysis of the peaks in the 2D HETCOR spectrum of HAP. Summary of the spectral properties discerned for the phosphates in each material is given in Table 2. Note that the percentage of the total signal for each species in this table is instantaneous and a more adequate representation of the relative intensity of each phosphate in each sample is provided by analysis of their dynamic behavior as a function of the CP contact time (see Table 3).

**Table 2:** Deconvolution results of one-dimensional ^31^P projections of HAP and HAP·qOPN.

Sample	δ (ppm)	Line width (ppm)	% of whole signal
**H**_**2**_**O**			
HAP	2.6	3.0	27
	3.3	5.6	73
HAP·qOPN	2.6	2.7	28
	3.4	5.4	72
**OH**^·^			
HAP	2.9	1.7	75
	3.3	3.5	25
HAP·qOPN	2.9	1.7	61
	3.3	3.3	39

Uncertainties in δ and line width are below ± 0.05 ppm and errors in relative signal intensities are < 5%.

**Table 3:** Kinetic parameters of the ^31^P magnetization CP buildup for HAP and HAP·qOPN.

HAP	Τ_cp_ (ms)	Τ_1ρ_ (ms)	I_o_	HAP·qOPN	Τ_cp_ (ms)	Τ_1ρ_ (ms)	I_o_
I	5.7 ± (0.1)	*	0.53 ± (0.08)	I	5.5 ± (0.1)	*	0.53 ± (0.07)
II	3.2 ± (0.3)	*	0.16 ± (0.02)	II	3.4 ± (0.2)	*	0.25 ± (0.04)
III	1.6 ± (0.3)	*	0.12 ± (0.02)	III	2.9 ± (0.4)	*	0.06 ± (0.02)
IV	1.3 ± (0.2)	7.9 ± (0.3)	0.19 ± (0.05)	IV	1.2 ± (0.3)	5.3 ± (0.2)	0.16 ± (0.03)

*The case where T_1ρ_^−1^ <  < T_cp_^−1^ and its contribution is therefore neglected.

This is further substantiated by examining the intensities of the cross peaks in a series of 2D ^1^H–^31^P HETCOR measurements recorded at variable CP contact times. The intensity of each of the phosphate species, in the two cross peaks, builds up at a different rate. Therefore, by analyzing the set of projections obtained through slicing, as before, along the H_2_O and OH^−^ resonances, the temporal intensity change per species is extracted (Fig. 4), for HAP·qOPN (blue symbols) and for HAP (red symbols). These experimental build-up curves are fitted to a theoretical CP buildup model (solid lines) using a home-written MATLAB minimization program.

1 $$I(t) = I_{0} \left( {1 - \frac{{T_{cp} }}{{T_{1\rho } }}} \right)^{ - 1} \cdot \left( {e^{{\frac{-t}{{T_{1\rho } }}}} - e^{{\frac{-t}{{T_{cp} }}}} } \right)$$

The kinetic parameters of the fitted model are summarized in Table 3. Equation 1 is used to fit the graphs with the parameters, $I_{0}$—the total magnetization of the phosphate species, $T_{cp}$—the effective magnetization buildup time (magnetization buildup occurs through ^1^H–^31^P dipolar couplings) and $T_{1\rho }$—the rotating-frame relaxation time which is usually taken as ^1^H $T_{1\rho }$ time since the proton relaxation process is faster and is hence the governing mechanism. This model assumes negligible spin diffusion between proton spins and that $T_{cp}$ is shorter than $T_{1\rho }$^59^. When $T_{1\rho } \gg T_{cp}$, the equation becomes simplified and independent of $T_{1\rho }$. This occurs for several phosphate species where an asterisk is written in Table 3 instead of a value for $T_{1\rho }$, indicating that the curve depends only on $I_{0}$ and $T_{cp}$. The total integrated intensity of the phosphate signal in HAP and HAP·qOPN is normalized to 1.

The normalized $I_{0}$ values give a crude assessment of the relative amount of phosphate species (excitable by cross polarization) in each sample. For comparison between samples, similar CP dynamics is not required here, however, since for some species a maximum intensity was not measured, the reliability in determination of $I_{0}$ is somewhat limited. Therefore, the comparison between intensities of different phosphates and relative sizes of phases (bulk, intermediate and surface) is not stringently quantitative. Under these restrictions, it is evident that the relative proportion of bulk:intermediate:surface in HAP·qOPN is 53:31:16 and that in HAP it is 53:28:19. The relative intensity of surface layer phosphates, excited via water protons (species IV), is slightly lower with the protein than without it. A similar phenomenon was also observed for HAP·umOC and for HAP·ON29^26,27^.

#### 2D ^31^P dipolar-assisted rotational resonance (DARR) experiment

**Figure 5:**
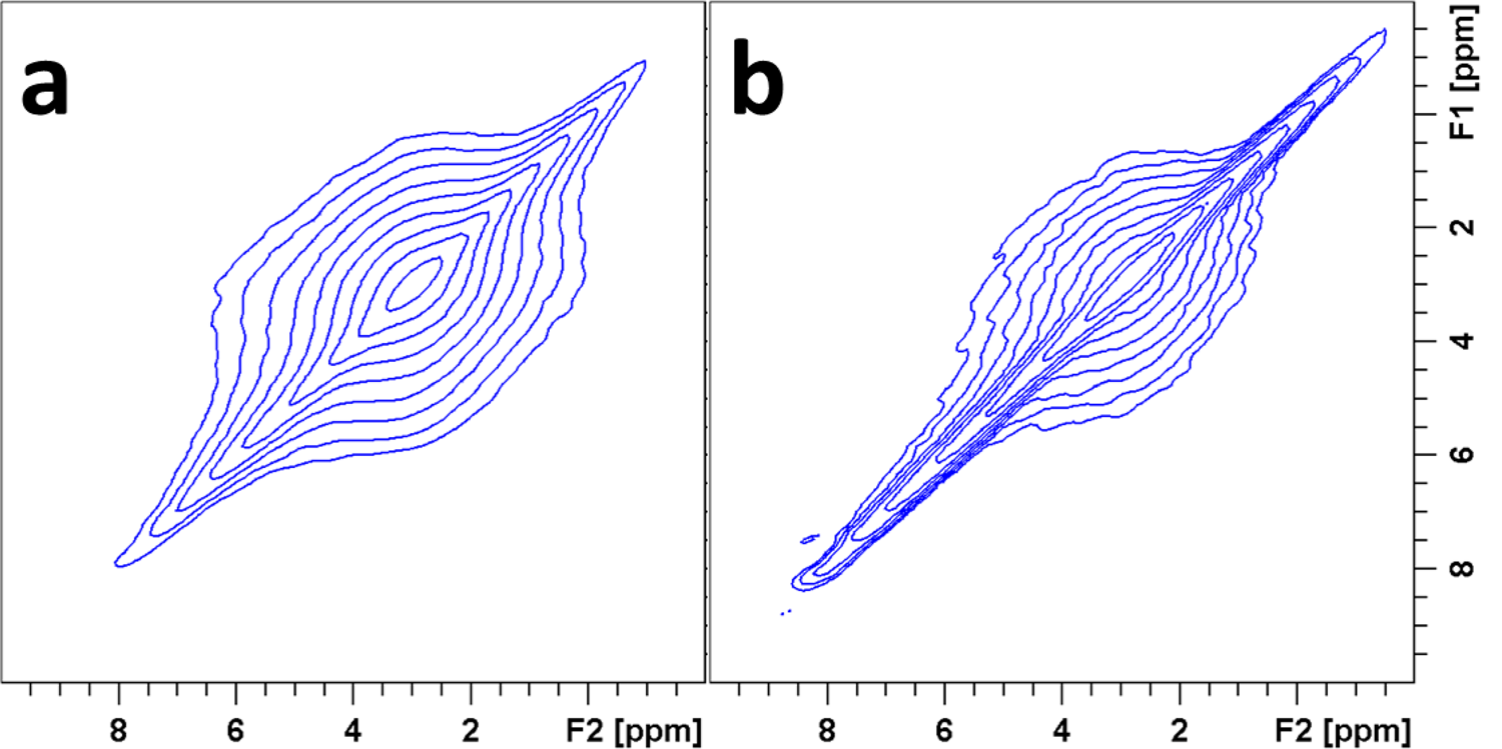
^31^P 2D DARR spectra of HAP (**a**) and HAP·qOPN (**b**) recorded using a contact time of 2 ms, a proton mixing time of 500 ms, 32 scans, and a recycle delay of 2 s.

Further evidence for protein effect on cross phase magnetization transfer between phosphates in the mineral is inferred from 2D ^31^P DARR experiment (Fig. 5). The phosphate diagonal peak is broader in HAP than in the mineral prepared with qOPN showing off-diagonal ridges from cross-species magnetization exchange extending to phosphate species at chemical shifts as high as 5.0 ppm. For the HAP·qOPN spectrum, the magnetization exchange is narrower involving mainly the disordered mineral at 3.2 ppm and up to ~ 3.5 ppm.

### NMR results of qOPN interactions with mineral phases

#### 1D CHHP relayed magnetization transfer experiments

The identification of disordered layers covering apatite crystallites also lead us to investigate qOPN location within these layers in HAP·qOPN. NMR measurements designed to detect phosphates at various distances from the carbon atoms in the protein, using the CHHP relayed transfer experiment^60^ (Fig. S10), were first employed. In the experiment, the ^31^P magnetizations of phosphates in the mineral phases are excited by qOPN ^13^C magnetizations, through various mediating hydrogens from the protein and from the minerals. During a designated proton mixing time ($\tau_{m}$) in the CHHP experiment, the protons are allowed to exchange magnetization between themselves, thereby enhancing transfer between distant ^13^C–^31^P pairs of nuclei via the stronger ^1^H–^1^H couplings. The protons, hence, facilitate indirect magnetization transfer pathways from ^13^C nuclei to more distant ^31^P nuclei and bypass the truncation of such pathways by closer ^31^P nuclei in direct transfer experiments such as TEDOR and double CP shown later.

**Figure 6:**
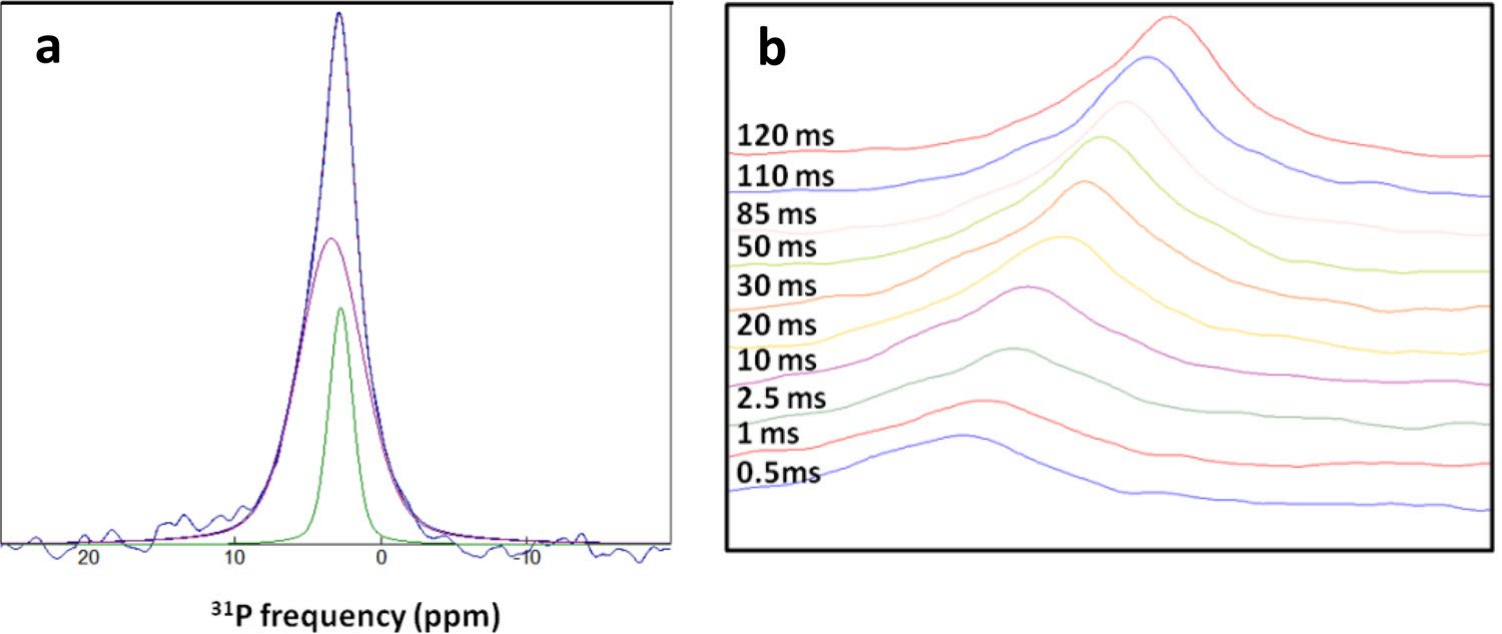
(**a**) 1D CHHP spectrum of HAP·qOPN (blue) recorded using HH mixing time of 120 ms. The line is comprised of two phosphate species, apatite phosphate (green line) and disordered phosphate (magenta line) obtained by deconvolution. (**b**) The buildup of phosphate magnetization in the 1D CHHP spectrum, given on the left, as a function of the HH mixing time, showing initial transfer to disordered PO_4_^3−^ and a gradual increase of the crystalline PO_4_^3−^ at longer mixing time.

The ^31^P CHHP spectrum of HAP·qOPN (Fig. 6a), obtained using a $\tau_{m}$ of 120 ms, shows a phosphate peak that comprises both disordered and crystalline phosphate species. The ^31^P spectra obtained at increasing $\tau_{m}$ are displayed in Fig. 6b, in a slanted view for clarity. At short mixing times, the spectra are broad comprising only disordered phosphates that obtain magnetization from carbons first. At longer mixing times the spectra are getting narrower and stronger and the phosphate peak shifts slightly to the left, indicating that crystalline phosphates are getting increasingly more magnetized.

**Figure 7:**
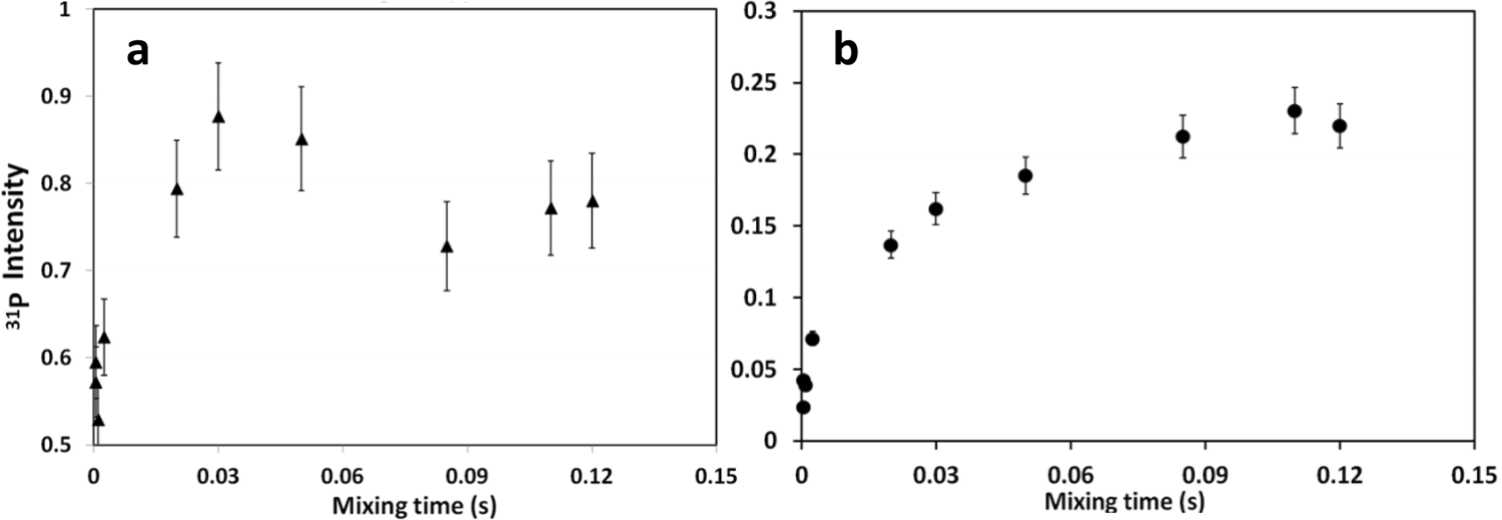
Buildup of ^31^P magnetization in 1D CHHP spectrum of HAP·qOPN as a function of the HH mixing time. (**a**) The disordered phosphate line buildup and (**b**) The apatite phosphate line buildup.

A plot of the phosphate magnetization intensity as a function of the mixing time, $\tau_{m}$, shows that the disordered phase ^31^P magnetization (Fig. 7a) reaches its maximal intensity after 30 ms, whereas crystalline phase ^31^P magnetization (Fig. 7b) reaches a plateau in its value at ~ 100 ms. Phosphates in the disordered mineral layers interact strongly with the protein carbons and obtain more than half of their maximal magnetization via a single mediating proton before any proton exchange processes were turned on. Apatite phosphates require magnetization to be relayed from protons on the protein to protons that are near the crystal surface, in a process with a longer characteristic time. A model to describe the transfer from ^13^C spins to the various ^31^P spins through the ^1^H “spin bath” is being constructed, but the qualitative comparison clearly indicates that the protein resides in closest proximity to the disordered phase phosphates and is farther removed from faces of the crystal, therefore, it is able to transfer magnetization to the core apatite phosphates only through protons that populate the intermediate layers closer to it and only at longer mixing times.

#### 2D ^31^P-^13^C double CP and ^13^C–^31^P zTEDOR experiments

**Figure 8:**
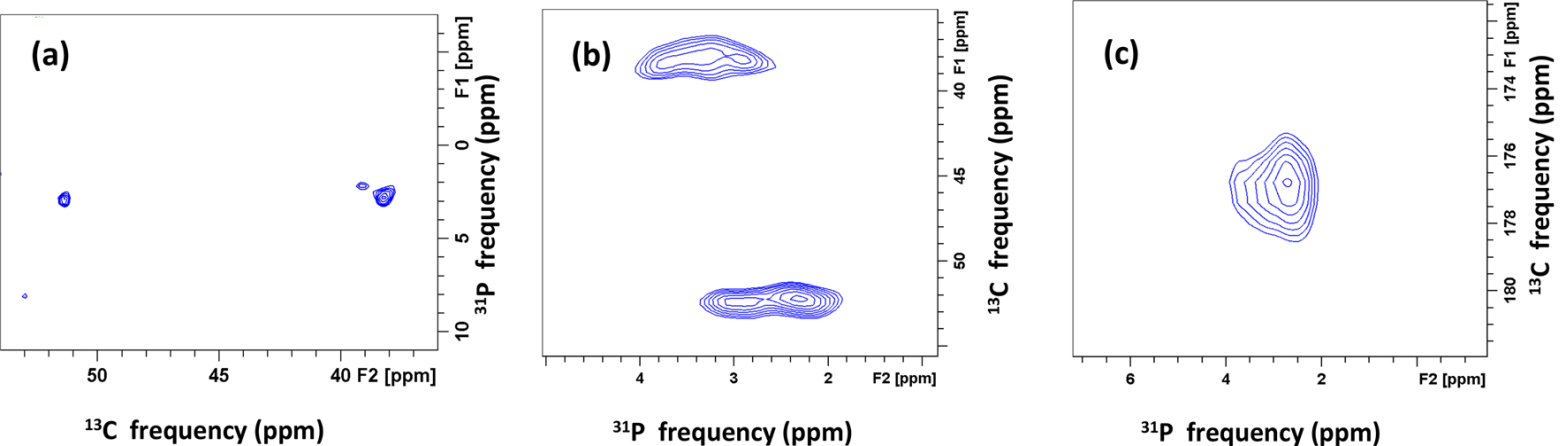
(**a**) 2D ^31^P-^13^C double-CP spectrum of HAP·qOPN with contact time of 5 ms between ^1^H and ^31^P, and 16 ms contact time between ^31^P and ^13^C showing only two cross peaks between apatite phosphate ions at 2.8 ppm and qOPN carbons. No cross peaks with the disordered phosphates are observed. (**b**) The aliphatic region and (**c**) the carbonyl region in the 2D rotor-synchronized ^13^C–^31^P z-filtered TEDOR spectrum of HAP·qOPN.

2D ^31^P-^13^C and ^13^C–^31^P correlation measurements of HAP·qOPN were also carried out to investigate direct transfer of magnetization from phosphates in the mineral to spatially proximate carbon atoms in qOPN molecules. The 2D ^31^P-^13^C DCP spectrum shown in Fig. 8a exhibits cross peaks between crystalline apatite phosphate resonance at 2.8 ppm (along the vertical axis) and carbon resonances at 51.4 ppm and at 38.3 ppm (along the horizontal axis), that, as a pair, can be assigned to Asn Cα and Asn Cβ, respectively. This measurement was carried using a ^31^P-^13^C CP contact time of 16 ms since shorter times did not produce cross peaks in the 2D spectra. The measurement suggests that only a single contact is observed between the apatite crystals and carbons in qOPN (entire spectrum is shown in Fig. S11).

The 2D ^13^C–^31^P z-filtered TEDOR^61^ spectra for the HAP·qOPN sample are shown in Fig. 8b (aliphatic region) and Fig. 8c (carbonyl region). Full z-filtered spectra are shown in Figs. S12 and S13. The cross peaks in the aliphatic region appearing at ~ 52 ppm and at ~ 38 ppm, correlate to the apatite phosphate resonance at 2.9 ppm. These correlations connect the same carbons to the apatitic phase as the cross peaks in ^31^P-^13^C DCP spectrum in Fig. 8a. The results, using two dipolar recoupling NMR techniques, confirm that only two types of aliphatic carbons in the whole protein are proximate to crystal surface of apatite and these are best associated with Asn Cα and Asn Cβ. Moreover, the carbonyl region spectrum (Fig. 8c) shows a strong cross peak at 176.8 ppm in ^13^C frequency with the apatite phosphate as well. This peak is not uniquely assigned, as it fits the frequency of carbonyls from various amino acids. However, it can also represent Asn Cγ, thereby, providing a more suitable view of the entire asparagine sidechain as being aligned very close to the crystal surface. Additional smaller cross peaks of carbonyl carbon (at ~ 177 ppm) with non-apatite phosphates are seen in Fig. 8c.

### Discussion

#### Influence of qOPN and other non-collagenous proteins on apatite crystallites

HRTEM data show that the mineral crystallites formed in HAP·qOPN, like in HAP·umOC^26^ and HAP·ON50^29^, have morphology and dimensions similar to that found in apatite platelets in bone, contrarily to HAP·ON29^27,28^, where needle-shaped crystallites are formed. The powder XRD data further corroborate the mild effect of qOPN on apatite crystallite properties compared to either ON29 or umOC. Though non-phosphorylated qOPN was reported to have no effect on apatite mineralization onset and crystal growth^32^, it is still interesting that it exhibits no marked changes to apatite crystallite characteristics, given its high content (24%) of Glu/Asp residues. The protein, however, has a marked effect on the disordered phases formed together with the apatite crystallites, observed via ICP and NMR measurements.

The dense crystallite aggregates observed in the scanning electron micrographs of HAP·qOPN relative to HAP resemble the ones recorded on HAP·ON29. They may be caused by strong adsorption forces of the biomolecules which can adhere to more than one crystallite thereby inducing aggregation by agglutination^27,28^. This also manifested in the lower SSA measured for HAP·qOPN than for HAP or for HAP·ON29. Moreover, the reported interactions between OPN molecules to form homodimers in addition to weaker dimer-monomer binding^62^ support the agglutination property and reduced SSA of qOPN-HAP as compared to OC and ON. Previous TGA measurements of HAP·ON29 showed that the peptide constitutes 4.8 weight% (1.6 mol%) of the material. Similarly, TGA of HAP·umOC showed that umOC constitutes 8.0 weight% of the material (1.4 mol%)^26,28^. Molar content of qOPN in the protein-mineral composite formed is much lower than ON29 or umOC. Interestingly, its pyrolysis is centered at a temperature 20 °C higher than umOC in HAP·umOC and ON29 in HAP·ON29, which asserts the stronger binding to the outer surfaces of apatite crystallites inferred from the SEM and specific surface area measurements.

The larger Ca/P ratio in the entire HAP·qOPN sample comprising the minerals and the protein, concurrently with hydroxyapatite being the only crystalline phase formed, indicates a depletion of phosphates from the non-crystalline phases detected by NMR. Similar Ca/P ratios were obtained in HAP·ON29 (1.82) and in an osteoporotic rib bone of a rabbit^63^. In HAP·umOC, Ca/P ratios were lower than HAP (1.56) indicating deficiency of Ca^2+^ ions from the disordered mineral phases as a result of the activity of umOC. Overall, variation of the ion composition in the amorphous mineral layers coating the apatite crystallites is one of the hallmarks of NCPs activity.

#### Identification of four different phosphate species

The 1D ^1^H–^31^P CP experiments suggests a well-ordered phosphates from the bulk of the apatite crystals excited by OH^−^ protons, denoted "species I", (peak at 2.9 ppm FWHM 1.7 ppm) and disordered phosphates from the disordered layer that covers apatite crystallites excited by water protons, denoted " species IV", (peak at 3.3–3.4 ppm FWHM 5.4–5.6) are commonly known and were identified by NMR before^9,10,58^. In addition, partially disordered phosphates excited by OH^−^ protons, denoted "species II", (peak at 3.3 ppm FWHM 3.3–3.5 ppm) and partially ordered phosphates excited by water protons, denoted "species III", (peak at 2.6 ppm FWHM 2.7–3.0 ppm) are also detected. The latter two *new* phosphate species, recognized recently^26,27^, define an intermediate mineral phase which resides spatially between the bulk apatite crystal and outer disordered phase. This phase contains PO_4_^3−^ and HPO_4_^2−^ ions characterized by transitional order (or disorder) compared to the crystalline and amorphous phases, as can be noticed from their intermediate linewidths.

It is evident from the intensities of the intermediate phase species that they constitute qualitatively, a significant proportion of the phosphates excitable by cross polarization in the samples. Yet, rigorous quantification of the absolute content of each phosphate species requires further scrutiny. The appearance of the phosphate at 2.6 ppm associated with protonated phosphate (HPO_4_^2−^) is interesting. It was observed before in HAP·umOC but not in HAP·ON29^26,27^. The proteins, qOPN and umOC, have similar isoelectric point of 4.2–4.4 and may therefore have a similar effect on the PO_4_^3−^ ⇌ HPO_4_^2−^ equilibrium, whereas the peptide with its more acidic pI of 3.2, maintains trivalent state of the ions.

The inter-phase phosphates (species III) have similar linewidth and proportionality out of the total disordered phosphate phase in HAP·qOPN like in HAP. The other inter-phase phosphates (species II), however, take up a larger fraction of the ordered phosphates with the protein as compared to HAP alone. The bulk phosphates (species I) linewidth is the same with qOPN and ON29 as in HAP and is slightly broader than in HAP·umOC, affirming that umOC is the only NCP that improves the order of these ions in the apatite crystal. The disordered phosphates in the surface layer (species IV) are marginally narrower with qOPN than in HAP alone or in HAP·umOC but are similar to preparation with ON29.

The interphase's disordered species (species II) in HAP·qOPN is slightly broader than in HAP·umOC and HAP·ON29, suggesting that phosphates closer to the outer surfaces of apatite crystals are strongly perturbed by the qOPN^26,27^. On the other hand, the interphase's ordered species (species III) in HAP·qOPN is narrower than other preparations indicating that qOPN induces an increase of ion ordering near the outer surface layer. Note that in HAP·ON29, this species appears at 2.8 ppm, however, it is not an apatitic phosphate, but rather a mix of partially ordered PO_4_^3−^ and HPO_4_^2−^ ion resonances that on average give a maximum peak at 2.8 ppm.

The bulk phosphates (species I) are characterized by high $T_{cp}$_-_value compared to the species from the other phases. This indicates that the rate of magnetization transfer to phosphorous atoms in the apatite crystal are the lowest, in accordance with the relatively long internuclear distances between OH^−^ protons and phosphate ^31^P nuclei in the lattice. The surface layer phosphates (species IV) show the lowest $T_{cp}$ values, 1.2 ms in HAP·qOPN and 1.3 ms in HAP indicating that they experience the strongest cumulative interaction with several protons. The interphase phosphates (species II and III) in HAP·qOPN, have similar $T_{cp}$, which is intermediate between the bulk and surface. In HAP, this parameter is quite different for the two species and suggests that qOPN’s presence in this layer may serve to balance the effective coupling experienced by phosphates close to faces of the crystal and close to the outer surface layer. This is unique to qOPN, as neither umOC^26^ nor ON29^27^ exhibit a similar effect on the interphase species. The $T_{cp}$ value of surface phosphates (species IV) in HAP·qOPN (1.2 ms) is close to value measured for fresh bone (1.1 ms)^10^.

The only species with measurable $T_{1\rho }$ time is the surface layer one (species IV). The smaller the value of $T_{1\rho }$ measured, the more effective are the motions that induce faster relaxation in the proton nuclei. For HAP·qOPN, this value is significantly smaller than for HAP, indicating that motions at the microsecond to millisecond time scale occur in the presence of the protein and these are efficiently promoting the faster relaxation detected. Whether this motions can be associated with hydrogen atoms in the protein, water molecules or protons on various ions requires further investigation, however, in the case of qOPN they produce lower $T_{1\rho }$ compared than measured with umOC^26^ and higher compared to values measured with ON29^27^ .

It is observed that the negatively charged protein, located in the disordered phases covering the apatite crystallites, decreases the content of phosphate species (PO_4_^3−^ and HPO_4_^2−^) in the disordered layer as observed by the ICP results as well. The I_0_ values are the relative populations of phosphate groups in different phases which allow us to estimate the Ca/P ratio in the surface and interphase jointly based on the ICP results and the stoichiometry of Ca/P in the apatite crystals. Ca/P ratio in the disordered phases was calculated for HAP·qOPN in the following way: Ca/P_(surface+interface)_*0.47 + 1.67*0.53 = 1.78, Ca/P_(surface+interface)_ = 1.90, taking the total relative intensity of the surface and interphase species from Table 3. The Ca/P_(surface+interface)_ value obtained for HAP is 1.65. The ion balance in the disordered phases, as can be seen, is influenced considerably by the negatively charged qOPN whereas the thickness of the interphase is only marginally affected. The phosphate depletion from the disordered phases with qOPN is, however, less extensive than with ON29^27^. Overall, it is evident that the NCPs affect the interphase differently, showing the regulatory capability over the minerals formed.

#### Interactions between different phosphate phases

The DARR measurement allows us to monitor slower spin diffusion processes and to observe magnetization transfer between phosphates that are farther apart. Here, it serves as a fingerprint for the extent of coupling between the crystalline phase (resonating at 2.8–2.9 ppm) and the amorphous phases, represented by phosphate peaks which are seen to extend all the way to 5.0 ppm. The de-shielded ^31^P nuclei in these amorphous phosphate species were typically invisible or buried under larger species in the standard ^31^P measurement. They represent minor populations of the ions that, in the presence of the protein molecules, shift downfield. In HAP, there is efficient transfer from the core crystallites to surface phosphates resonating at 5.0 ppm, whereas, in HAP·qOPN the transfer is limited between crystalline phosphates and phosphates at 3.5 ppm, characterized by a relatively narrow line width.

#### Confirmation of location of qOPN in interfacial disorder mineral phases

The CHHP measurement shows that the qOPN molecules are located in disordered mineral layers covering the apatite crystallites and they exert activity from this location. This also explains the stronger influence of the protein on the properties of the disordered hydrated layers than on apatite crystallites. qOPN is able to alter the composition of mineral ions in this layer, pushing more Ca^2+^ ions to accommodate the layer than phosphate ions. It affects also the dynamic properties of the water and phosphate ions in this layer as compared to the properties of these moieties in HAP, as inferred from the CP buildup experiments shown above.

In addition, the higher sensitivity in the TEDOR measurements reveals that Asn Cβ carbon at ~ 38 ppm is correlated with two more phosphate species centered at ~ 3.3 ppm and at ~ 3.8 ppm. In addition, the Asn Cα carbon is correlated to a phosphate species at ~ 2.3 ppm along the ^31^P axis. The linewidths of these phosphates are evidently similar to the linewidth of the apatite line, suggesting that they are associated with highly ordered phosphate ions. Their chemical shifts match the shifts of phosphates P1 (3.8 ppm), P4 (3.3 ppm) and P2 (2.0 ppm) in crystalline octacalcium phosphate (OCP). Out of the six unique phosphates in the crystal lattice of OCP, these three are closer to the apatite layer^64^. It is quite compelling to observe the interaction of specific carbons in the Asn residue with crystallographically unique phosphates in OCP and in any mineral in general. This would be hard to observe in hydroxyapatite which has its three unique phosphates appearing at the same chemical shift^65^.

The scarcity of cross peaks between the protein and crystalline apatite phosphates is consistent with the CHHP results and asserts the presence of most of the protein away from the crystallite surface with only a single type of residue in direct interaction with it. At the same time, the absence of cross peaks between qOPN carbons and disordered phosphates is less obvious, since CHHP data indicated residence of the protein in the disordered layers. This can be explained by motional averaging of phosphates in these layers and/or protein sidechains that efficiently averages the direct magnetization transfer between disordered phosphates and qOPN carbons but is ineffective in case of the relayed transfer through the adjacent protons. The phosphates in the coat layers were shown above to experience motions. Other measurements, which will be reported elsewhere, indicate that the protein retains considerable amount of dynamics in the mineral-bound state as well.

The common paradigm of the bio-inorganic interface, formed between OPN, as well as other non-collagenous proteins, with bone apatite and other important biominerals, assumes binding of the protein mineral to flat well-ordered faces of apatite crystals^66^. Other visualizations of the interaction using molecular dynamics simulations, interestingly, have shown OPN binds intermittently to surfaces of crystals while retaining some of its intrinsic dynamic behavior as an IDP^36^. However, none of the current models, accounts for the observation of non-vanishing disordered layers coating the exposed faces of the apatite crystallites.

**Figure 9:**
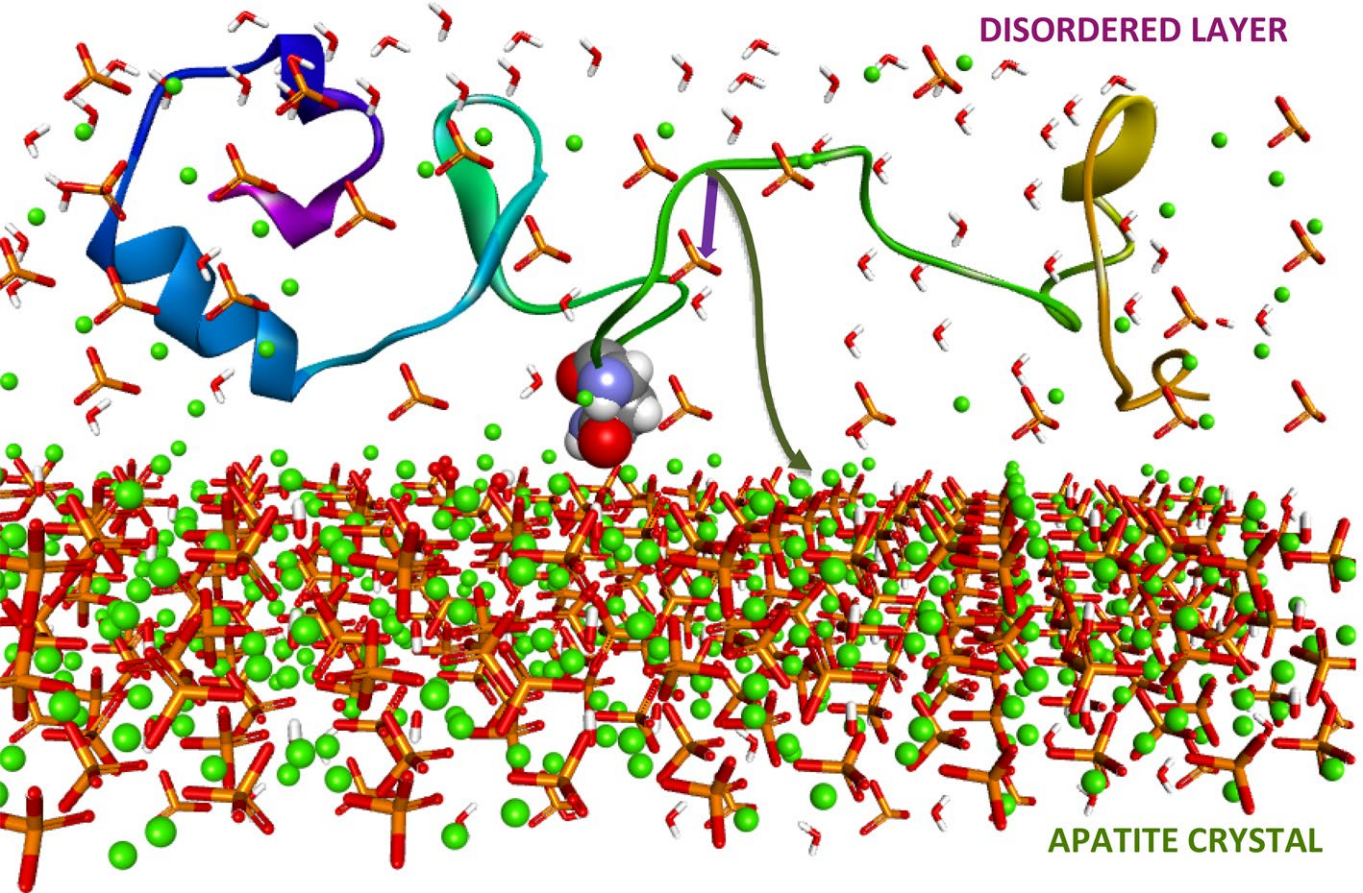
NMR-based model showing qOPN (structure predicted using the ROSETTA algorithm^67^) located in a disordered layer covering the apatite crystal and having Asn sidechain able to reach in and interact with the surface of the crystal. Purple and green arrows represent magnetization transfers first to disordered PO_4_^3−^ ions and later to apatite PO_4_^3−^ ions.

Given the observed protein-mineral interactions in this work, predominantly through the disordered layers, the view of the direct atomic interface between protein molecules and mineral ions may need to be modified. A revised view of the mineral present at the bio-inorganic interface, shown in Fig. 9, which includes the persistent disordered phases of calcium encompassing phosphate ions with which the protein interacts predominantly. Residence in a disordered mineral phase may preserve some of the protein's solution motions, since the mineral ions themselves, are unconstrained by the crystal field in such layers, and thus may be experiencing motions.

Activity of functional proteins in such environment, crowded by inorganic ions, that is quite different from the typically considered cellular environment, crowded mostly with other biomacromolecules, is interesting and may require modification of the potentials and forcefields used to model their structure, dynamics and binding energy. Proteins with pronounced motional characteristics in solution such as intrinsically disordered proteins, especially ones with polyproline segments, might be able to retain some of their dynamics in the crowded regions such as disordered mineral phases. The view of OPN as a flexible electrolyte may still hold for protein molecules embedded in disordered mineral layers, however, the denser environment of ions needs to be accounted for in modeling these motions.

The involvement of at least a single residue sidechain in direct interaction with the crystal face of apatite and OCP indicates that for qOPN and perhaps for other NCPs, contact with the crystalline phase is maintained even if it is quite subtle. Binding to specific crystal faces (face recognition) through multiple contact points and control over crystal growth directions and morphology was not observed here for non-phosphorylated version of quail osteopontin, however, it may represent the functional mechanism of phosphorylated OPN and its fragments as well as other NCPs. The use of a high-resolution technique such as solid-state NMR exemplifies the importance of having an atomic view of the interaction interface experimentally to appreciate the complexity of mechanisms underlying the end-product biomaterials that are generated in organisms.

## Conclusions

OPN is shown to have an impact on the mineralization of apatite at physiological temperature under controlled conditions. The mineral is precipitated with as much as a quarter mol% of the protein. The core apatitic crystallites preserve their platelet-like morphology but tend to agglomerate in the presence of OPN, suggesting an agglutinating property for the protein. NMR measurements show that an appreciable disordered calcium phosphate mineral layer covers the apatite crystallites. From quantification of Ca and P in HAP·qOPN, it is clear that the surface layer is depleted of phosphate groups. Solid state NMR techniques allow further identification of various disordered phases^68,69^. A partially ordered mineral inter-phase layer between the ordered and disordered phases is observed in HAP·qOPN as was shown recently for other bone proteins. The magnetization transfer dynamics proves to be a sensitive tool to trace the influence of qOPN on the disordered mineral phases that form with the apatite crystallites and to elaborate on its distinct effects compared to other bone proteins. Finally, it is shown for the first time through the CHHP, 2D ^31^P-^13^C DCP and 2D ^13^C–^31^P z-filtered TEDOR experiments that the protein resides in the disordered coating layers with one or few sidechains at most reaching the faces of the apatite crystallites and therefore exerts its regulating function on the crystals formed from a distance.

Understanding the binding and activity of qOPN as a key NCP in regulation of apatite homeostasis, gives leads to how properties of other mineralized materials are regulated. The formation of an interphase between a crystalline phase and a disordered layer (aka amorphous calcium phosphate) and the ability of NCPs to regulate the composition of disordered phases may be a common mechanism of mineral regulation and may have ramifications for the mechanical properties of bone mineral. The NMR analyses demonstrated in this work can help understand the pathological impact of lack or deficiency of key biomolecules such as osteopontin on mineralization processes.

## Experimental

### Materials

Di-ammonium hydrogen phosphate (NH_4_)_2_HPO_4_ 99% and calcium nitrate tetrahydrate Ca(NO_3_)_2_·4H_2_O 99% were purchased from Merck and Sigma Aldrich, respectively. Ammonium hydroxide NH_4_OH was purchased from BioLab Ltd.

Bioexpression of quail OPN was carried out similarly to procedure reported recently^70^. Coturnix japonica (or quail) OPN DNA sequence was cloned into a pET11d plasmid and was then transformed into a phage resistant E. coli strain BL21(DE3) via a heat-shock technique. Expression of the protein was induced at OD_600_ of approximately 0.8 by addition of 0.4 mM IPTG (Isopropyl-β-D-Thiogalactopyranoside). To express the protein with isotope labels for NMR measurements, the cells were harvested before induction and the cell pellets from growth in 4 L with LB were resuspended in 1 L M9-Minimal Media (containing 3 g/L ^12^C-Glucose and 1 g/L ^15^NH4Cl for ^15^ N labelling). The next step of expression was performed at 27 °C and 140 rpm over-night. The cells were, then, harvested and resuspended in 40 mL of cold PBS (2 mM KH_2_PO_4_, 8 mM Na_2_HPO_4_, 2.5 mM KCl, 140 mM NaCl, 5 mM EDTA, pH 7.3) prior to cell lysis. The suspended cells were sonicated and warmed to 95 °C and kept at that temperature for 10 min. The lysate was spun down at 18,000 rpm and ammonium sulfate precipitation (saturation of 50%) was carried out with the supernatant. The pellet was, finally, resuspended in PBS, diluted 1:2 with water to lower the salt concentration and the protein was separated by anion-exchange chromatography using a HiTrap Q column from GE healthcare. The column was equilibrated with PBS and a gradient of 30% High-Salt Buffer (PBS containing 1 M NaCl) was run for 20 min at a flow rate of 2 mL/min. The protein was exchanged to PBS buffer at pH 6.5 and concentrated using a centrifugal filter and the final concentration was measured by absorbance at 280 nm. Protein purity was confirmed via SDS-PAGE. Quail osteopontin has a molecular weight of 24.3 kDa and a calculated pI of 4.6.

### Methods

#### Synthesis of apatite in the presence ^15^ N qOPN

Synthesis of the mineral in the presence of the protein was performed according to procedures published recently^27,28^. Briefly, hydroxyapatite was synthesized at 37 °C and pH 9 in the presence of [U-^15^ N(95%)]qOPN (denoted as ^15^ N qOPN) by titrating 16 ml of 0.075 M (NH_4_)_2_HPO_4_ into 56 ml of 0.040 M calcium nitrate and 0.2 M (0.8%) ammonium hydroxide solution in which 42.8 mg of peptide were dissolved. Synthesis without ^15^ N qOPN followed identical conditions without addition of the protein into the Ca(NO_3_)_2_-NH_4_OH solution. All syntheses were carried out under air. The titration was performed over duration of 5 h at a rate of 0.053 ml/min using a peristaltic pump. Solution was stirred at a rate of 156 rpm with temperature held constant and pH corrected every 5 min by adding small aliquots of 24% ammonium hydroxide solution as necessary. After the phosphate solution was added, the temperature was set to 60 °C and the mixture stirred overnight. The solid precipitate was filtered and rinsed with double distilled water, 18.2 MΩ (DDW). Excess water was removed from sediment by leaving the samples at 37 °C for 60 h. HAP and HAP·^15^ N qOPN crystals were pulverized in a porcelain mortar and stored as is. Similar procedures were used for synthesis of reference hydroxyapatite (without peptide).

#### Powder X-Ray Diffraction and crystallite size analysis

Powder X-ray diffractograms were recorded using a Cu Kα radiation source (1.54 Å) on a Bruker AXS D8 Advance diffractometer. Measurements were performed on similar quantities of specimens in a 2θ range of 20°-80° for a duration of 0.05 s. Size of crystallites were calculated using the Scherer equation with an angle of 25.9°, λ = 1.542 Å, a shape factor of 0.9 and using the reflection linewidth in the diffractograms.

#### Inductively coupled plasma (ICP) analysis

ICP analysis was performed using ULTIMA2 device from Jovin-Yvon-Horiba. Quantification of elemental calcium and phosphorous content in the materials was carried out by suspending 1 mg of product powder in 10 ml of 0.1 M HCl before injection to the plasma source. Relative errors in Ca and P measurements were used to determine errors in Ca/P ratios. Relative error in these ratios for the two elements are between 0.65% and 1.07%. ICP measurements of apatite mineral precipitated with other NCPs such as osteocalcin were done giving Ca/P lower than 1.6 (data not shown) to verify that there is no bias the device used for the measurement in this work.

#### Elemental analysis

For carbon, hydrogen, nitrogen and sulfur elemental content analysis, 8 mg of sample was introduced into a Thermo FlashEA 1112 Series analyzer.

#### Brunauer–Emmett–Teller isotherm (BET)

Surface area was measured on a NOVA 3200E Quantachrome Instruments surface area and pore size analyzer. Similar values of surface area were recorded for HAP, 69.2 mg and for HAP·qOPN, 69.4 mg.

#### Transmission and Scanning Electron Microscopy (TEM and SEM)

High-resolution TEM measurements were carried out on a JEM 2100, JEOL instrument with LaB6 e-beam source at acceleration voltages of 200 kV. Surface analysis of specimens were carried out on a FEI, Magellan 400L high-resolution SEM instrument. Prior to TEM measurements, specimens were immersed in ethanol, sonicated for 10 min and placed on copper grids until solvent had evaporated. For HRSEM, samples suspended in water were deposited on a double-sided 12 mm thick carbon tape.

#### Thermogravimetric Analysis (TGA)

TGA measurements were carried out using 5 mg of sample on a TGA Q500 TA Instruments analyzer. The temperature was first increased from room temperature at a heating rate of 10 °C/min to 120 °C, and left to anneal at 120 °C for 30 min. The weight loss was measured with a similar temperature gradient to 950 °C.

#### NMR experiments

HAP·^15^ N qOPN and HAP samples were packed into 4 mm MAS NMR rotors. Most solid-state NMR experiments were carried out at room temperature on an 11.74 T Bruker Avance III spectrometer equipped with a 4 mm MAS VTN probe. The 2D ^13^C–^31^P double CP experiments were performed on a 16.4 T Bruker Avance III spectrometer at Bruker Biospin in Rheinstetten, Germany. ^31^P direct polarization (DP) experiments were performed using spinning rate of 10 kHz, 2.5 µs ^31^P 90° pulse and recycle delay of 40 s. ^31^P CPMAS experiments were carried out using a spinning rate of 10 kHz, a 2.6 µs ^1^H 90° pulse, 3 ms contact pulse, SPINAL64 pulse scheme for proton decoupling^71,72^ and recycle delay of 1 s. Slow spinning ^31^P CPMAS experiments were performed at a spinning rate of 2 kHz to extract CSA parameters. ^1^H–^31^P HETCOR experiments were performed using spinning rate of 10 kHz, a 2.55 µs ^1^H 90° pulse, CP contact pulse of 3.5 ms and the PMLG5 scheme for ^1^H homonuclear decoupling at an effective field of 120 kHz during t_1_^73,74^. The two-pulse phase modulation TPPM pulse scheme was used for proton decoupling and the field used was 98 kHz^75^. All HETCOR experiments employed a recycle delay of 0.7 s. ^31^P chemical shifts were calibrated relative to H_3_PO_4_ (85%). Signal to noise values in 2D HETCOR experiments were used as the basis for error bar calculations in CP build-up curves. 1D ^31^P NMR data were processed using a 20 Hz line broadening exponential multiplication resulting in line widths that are 0.1 ppm broader than the natural line width of peaks in the spectrum.

2D ^31^P–^31^P DARR measurements were performed at 10 kHz using 2.5 µs ^1^H 90° pulse, contact pulse of 2 ms, 500 ms of mixing time, SPINAL64 ^1^H homonuclear decoupling and recycle delay of 2 s.

1D (^31^P detected) CHHP measurements^54^ were performed using spinning rate of 10 kHz, 2.75 μs ^1^H 90° pulse, 2 ms contact pulse between ^1^H and ^13^C in first and second CP periods, 5.3 μs ^13^C 90° pulse, 20 ms z-filter time, 3 ms ^1^H–^31^P cross polarization time, SPINAL64 ^1^H homonuclear decoupling at a field of 80 kHz with a recycle delay of 1 s.

2D ^31^P-^13^C double-CP experiments were performed at a spinning rate of 31 kHz with an initial ^1^H–^31^P contact pulse of 5 ms using a ramped lock pulse on ^1^H between 58 and 83 kHz and a lock field of 36 kHz on ^31^P, followed by a ^31^P-^13^C contact pulse of 16 ms, using a ramped lock field on the ^13^C between 36 and 40 kHz and a lock field of 71 kHz on ^31^P. A total of 24 points of ^31^P evolution were collected in t_1_ with 16 k repetitions of ^13^C acquisition and a recycle delay of 2 s. Processing of 2D involved no linear back-prediction, only exponential window function with 0.3 Hz and 20 Hz in F1 and F2 dimensions, respectively.

Rotor synchronized 2D ^31^P-^13^C z-filtered TEDOR experiments were performed at a spinning rate of 10 kHz with an initial ^1^H–^31^P CP contact pulse of 2.5 ms and using 12 μs ^13^C 180° pulses and 8 μs ^31^P 180° pulses and twice shorter respective 90° pulses on the two channels. Each of the two TEDOR recoupling periods in the experiment were 4.8 ms long. The z-filter delays employed 10 kHz irradiation on the protons for a duration of 2 ms each. Overall, 50 t_1_ points were collected in the indirect dimension with 2048 repetitions and a recycle delay of 2 s.

#### NMR simulations and data fitting

Chemical shift anisotropy of 14.8 kHz and asymmetry of 1 were deduced from slow spinning ^31^P CPMAS measurements by fitting the sideband patterns to simulated ones obtained using SIMPSON^76^. These chemical shift parameters are similar to values measured for HAP before. Deconvolutions of ^31^P lines were carried out using the DMFIT program developed by Massiot and co-workers^57^.

## Supplementary information

Supplementary information

## References

[1] Mahamid J (2010). Mapping amorphous calcium phosphate transformation into crystalline mineral from the cell to the bone in zebrafish fin rays. Proc. Natl. Acad. Sci..

[2] Rey C, Combes C, Drouet C, Glimcher M (2009). Bone mineral: update on chemical composition and structure. Osteoporos. Int..

[3] Mroue KH (2012). High-resolution structural insights into bone: a solid-state NMR relaxation study utilizing paramagnetic doping. J. Phys. Chem. B.

[4] Mroue KH (2014). Acceleration of natural-abundance solid-state MAS NMR measurements on bone by paramagnetic relaxation from gadolinium-DTPA. J. Magn. Reson..

[5] Mroue KH (2015). Proton-detected solid-state NMR spectroscopy of bone with ultrafast magic angle spinning. Sci.Rep..

[6] Xu J (2010). Natural-abundance 43Ca solid-state NMR spectroscopy of bone. J. Am. Chem. Soc..

[7] Zhu P (2009). Time-resolved dehydration-induced structural changes in an intact bovine cortical bone revealed by solid-state NMR spectroscopy. J. Am. Chem. Soc..

[8] Reznikov N, Bilton M, Lari L, Stevens MM, Kröger R (2018). Fractal-like hierarchical organization of bone begins at the nanoscale. Science.

[9] Wang Y (2012). The predominant role of collagen in the nucleation, growth, structure and orientation of bone apatite. Nat. Mater..

[10] Wang Y (2013). Water-mediated structuring of bone apatite. Nat. Mater..

[11] Tsortos A, Nancollas GH (2002). The role of polycarboxylic acids in calcium phosphate mineralization. J. Colloid Interface Sci..

[12] Sugino A, Miyazaki T, Ohtsuki C (2008). Apatite-forming ability of polyglutamic acid hydrogels in a body-simulating environment. J. Mater. Sci. Mater. Med..

[13] Wazen RM (2007). In vivo functional analysis of polyglutamic acid domains in recombinant bone sialoprotein. J. Histochem. Cytochem..

[14] Nudelman F (2010). The role of collagen in bone apatite formation in the presence of hydroxyapatite nucleation inhibitors. Nat. Mater..

[15] Fujisawa R, Kuboki Y (1992). Affinity of bone sialoprotein and several other bone and dentin acidic proteins to collagen fibrils. Calcif. Tissue Int..

[16] Neary MT (2011). Contrasts between organic participation in apatite biomineralization in brachiopod shell and vertebrate bone identified by nuclear magnetic resonance spectroscopy. J. R. Soc. Interface.

[17] Rodriguez DE (2014). Multifunctional role of osteopontin in directing intrafibrillar mineralization of collagen and activation of osteoclasts. Acta Biomater..

[18] Hu Y, Rawal A, Schmidt-Rohr K (2010). Strongly bound citrate stabilizes the apatite nanocrystals in bone. Proc. Natl. Acad. Sci. USA.

[19] Davies E (2014). Citrate bridges between mineral platelets in bone. Proc. Natl. Acad. Sci. USA.

[20] Reid D (2013). Citrate occurs widely in healthy and pathological apatitic biomineral: mineralized articular cartilage, and intimal atherosclerotic plaque and apatitic kidney stones. Calcif. Tissue Int..

[21] Liu Y, Luo D, Wang T (2016). Hierarchical structures of bone and bioinspired bone tissue engineering. Small.

[22] Hunter GK, Hauschka PV, Poole AR, Rosenberg LC, Goldberg HA (1996). Nucleation and inhibition of hydroxyapatite formation by mineralized tissue proteins. Biochem. J..

[23] Sodek J, Ganss B, McKee M (2000). Osteopontin. Crit. Rev. Oral Biol. Med..

[24] Arachchige RJ (2018). Solid-state NMR identification of intermolecular interactions in amelogenin bound to hydroxyapatite. Biophys. J..

[25] Du Y-P (2016). Study of binding interaction between Pif80 protein fragment and aragonite. Sci. Rep..

[26] Iline-Vul T (2019). How does osteocalcin lacking γ-glutamic groups affect biomimetic apatite formation and what can we say about its structure in mineral-bound form?. J. Struct. Biol..

[27] Iline-Vul T, Matlahov I, Grinblat J, Keinan-Adamsky K, Goobes G (2015). Changes to the disordered phase and apatite crystallite morphology during mineralization by an acidic mineral binding peptide from osteonectin. Biomacromolecules.

[28] Matlahov I (2015). Interfacial mineral-peptide properties of a mineral binding peptide from osteonectin and bone-like apatite. Chem. Mater..

[29] Iline-Vul T (2018). Understanding the roles of functional peptides in designing apatite and silica nanomaterials biomimetically using NMR techniques. Curr. Opin. Colloid Interface Sci..

[30] George A, Veis A (2008). Phosphorylated proteins and control over apatite nucleation, crystal growth, and inhibition. Chem. Rev..

[31] Lenton S (2017). Effect of phosphorylation on a human-like osteopontin peptide. Biophys. J..

[32] Gericke A (2005). Importance of phosphorylation for osteopontin regulation of biomineralization. Calcif. Tissue Int..

[33] O’Regan AW (1999). Osteopontin is associated with T cells in sarcoid granulomas and has T cell adhesive and cytokine-like properties in vitro. J. Immunol..

[34] Agnholt J (2007). Osteopontin, a protein with cytokine-like properties, is associated with inflammation in Crohn's disease. Scand. J. Immunol..

[35] Morinobu M (2003). Osteopontin expression in osteoblasts and osteocytes during bone formation under mechanical stress in the calvarial suture in vivo. J. Bone Miner. Res..

[36] Hunter GK, O’Young J, Grohe B, Karttunen M, Goldberg HA (2010). The flexible polyelectrolyte hypothesis of protein–biomineral interaction. Langmuir.

[37] Hunter GK (2013). Role of osteopontin in modulation of hydroxyapatite formation. Calcif. Tissue Int..

[38] Ravindran S, George A (2014). Multifunctional ECM proteins in bone and teeth. Exp. Cell Res..

[39] Addison WN, Masica DL, Gray JJ, McKee MD (2010). Phosphorylation-dependent inhibition of mineralization by osteopontin ASARM Peptides Is regulated by PHEX cleavage. J. Bone Miner. Res..

[40] Martin A (2008). Degradation of MEPE, DMP1, and release of SIBLING ASARM-Peptides (Minhibins): ASARM-peptide(s) are directly responsible for defective mineralization in HYP. Endocrinology.

[41] Li SY, Wang LJ (2012). Phosphorylated osteopontin peptides inhibit crystallization by resisting the aggregation of calcium phosphate nanoparticles. CrystEngComm.

[42] de Bruyn JR (2013). Dynamic light scattering study of inhibition of nucleation and growth of hydroxyapatite crystals by osteopontin. PLoS ONE.

[43] Li SY, Wu SS, Nan DF, Zhang WJ, Wang LJ (2014). Inhibition of pathological mineralization of calcium phosphate by phosphorylated osteopontin peptides through step-specific interactions. Chem. Mater..

[44] Klaning E, Christensen B, Sorensen E, Vorup-Jensen T, Jensen J (2014). Osteopontin binds multiple calcium ions with high affinity and independently of phosphorylation status. Bone.

[45] Pampena D (2004). Inhibition of hydroxyapatite formation by osteopontin phosphopeptides. Biochem. J..

[46] Silverman L (2010). Hydroxyapatite growth inhibition by osteopontin hexapeptide sequences. Langmuir.

[47] Fisher LW, Torchia DA, Fohr B, Young MF, Fedarko NS (2001). Flexible structures of SIBLING proteins, bone sialoprotein, and osteopontin. Biochem. Biophys. Res. Commun..

[48] Dyson HJ, Wright PE (2005). Intrinsically unstructured proteins and their functions. Nat. Rev. Mol. Cell Biol..

[49] Boskey AL, Villarreal-Ramirez E (2016). Intrinsically disordered proteins and biomineralization. Matrix Biol..

[50] Kurzbach D (2013). Cooperative unfolding of compact conformations of the intrinsically disordered protein osteopontin. Biochemistry.

[51] Konrat R (2014). NMR contributions to structural dynamics studies of intrinsically disordered proteins. J. Magn. Reson..

[52] Platzer G (2011). The metastasis-associated extracellular matrix protein osteopontin forms transient structure in ligand interaction sites. Biochemistry.

[53] Thurner PJ (2010). Osteopontin deficiency increases bone fragility but preserves bone mass. Bone.

[54] Poundarik AA (2012). Dilatational band formation in bone. Proc. Natl. Acad. Sci. USA.

[55] Nikel OE, Laurencin D, McCallum SA, Gundberg CM, Vashishth D (2013). NMR investigation of the role of osteocalcin and osteopontin at the organic–inorganic interface in bone. Langmuir.

[56] Ibsen CJS, Gebauer D, Birkedal H (2016). Osteopontin stabilizes metastable states prior to nucleation during apatite formation. Chem. Mater..

[57] Massiot D (2002). Modelling one- and two-dimensional solid-state NMR spectra. Magn. Reson. Chem..

[58] Jäger C, Welzel T, Meyer-Zaika W, Epple M (2006). A solid-state NMR investigation of the structure of nanocrystalline hydroxyapatite. Magn. Reson. Chem..

[59] Mathew R (2011). Solid-state 31P and 1H NMR investigations of amorphous and crystalline calcium phosphates grown biomimetically from a mesoporous bioactive glass. J. Phys. Chem. C.

[60] Morag O, Abramov G, Goldbourt A (2014). Complete chemical shift assignment of the ssDNA in the filamentous bacteriophage fd reports on its conformation and on its interface with the Capsid Shell. J. Am. Chem. Soc..

[61] Jaroniec CP, Filip C, Griffin RG (2002). 3D TEDOR NMR experiments for the simultaneous measurement of multiple carbon–nitrogen distances in uniformly 13C, 15N-labeled solids. J. Am. Chem. Soc..

[62] Goldsmith H (2002). Homotypic interactions of soluble and immobilized osteopontin. Ann. Biomed. Eng..

[63] Kourkoumelis N, Balatsoukas I, Tzaphlidou M (2012). Ca/P concentration ratio at different sites of normal and osteoporotic rabbit bones evaluated by Auger and energy dispersive X-ray spectroscopy. J. Biol. Phys..

[64] Davies E (2014). Ca/P concentration ratio at different sites of normal and osteoporotic rabbit bones evaluatedCitrate bridges between mineral platelets in bone. Proc. Natl. Acad. Sci. USA.

[65] Raghunathan V (2006). Homonuclear and heteronuclear NMR studies of a statherin fragment bound to hydroxyapatite crystals. J. Phys. Chem. B.

[66] Athanasiadou D (2018). Nanostructure, osteopontin, and mechanical properties of calcitic avian eggshell. Sci. Adv..

[67] Rohl CA, Strauss CEM, Misura KMS, Baker D (2004). Protein structure prediction using rosetta. Numer. Comput. Methods Pt D.

[68] Roehrich A, Drobny G (2013). Solid-state NMR studies of biomineralization peptides and proteins. Acc. Chem. Res..

[69] Shaw W (2015). Solid-state NMR studies of proteins immobilized on inorganic surfaces. Solid State Nucl. Magn. Reson..

[70] Mateos B (2020). The ambivalent role of proline residues in an intrinsically disordered protein: from disorder promoters to compaction facilitators. J. Mol. Biol..

[71] Fung B, Khitrin A, Ermolaev K (2000). An improved broadband decoupling sequence for liquid crystals and solids. J. Magn. Reson..

[72] Brauniger T, Wormald P, Hodgkinson P (2002). Improved proton decoupling in NMR spectroscopy of crystalline solids using the SPINAL-64 sequence. Monatshefte Fur Chemie.

[73] Vinogradov E, Madhu P, Vega S (2000). A bimodal Floquet analysis of phase modulated Lee-Goldburg high resolution proton magic angle spinning NMR experiments. Chem. Phys. Lett..

[74] Vinogradov E, Madhu P, Vega S (1999). High-resolution proton solid-state NMR spectroscopy by phase-modulated Lee-Goldburg experiment. Chem. Phys. Lett..

[75] Bennett A, Rienstra C, Auger M, Lakshmi K, Griffin R (1995). Heteronuclear decoupling in rotating solids. J. Chem. Phys..

[76] Bak M, Rasmussen J, Nielsen N (2000). SIMPSON: A general simulation program for solid-state NMR spectroscopy. J. Magn. Reson..

