# Supplementary material for: Osteopontin regulates biomimetic calcium phosphate crystallization from disordered mineral layers covering apatite crystallites
Source: Sci Rep. 2020 Sep 24;10:15722. doi: 10.1038/s41598-020-72786-x (PMC7518277; doi:10.1038/s41598-020-72786-x)
Supplement: Supplementary file 1 — Supplementary information [file 41598_2020_72786_MOESM1_ESM.docx]

**Supporting Information**

**Osteopontin Regulates Biomimetic Calcium Phosphate Crystallization from Disordered Mineral Layers Covering Apatite Crystallites**

Taly Iline-Vul^1^, Raju Nanda^1^, Borja Mateos^2^, Shani Hazan^1^, Irina Matlahov^1^, Ilana Perelshtein^1^, Keren Keinan-Adamsky^1^, Gerhard Althoff-Ospelt^3^, Robert Konrat^2^, Gil Goobes^1*^

*^1^Department of Chemistry, Bar Ilan University Ramat Gan 52900, Israel*

*^2^Max F. Perutz Laboratories, Department of Computational and Structural Biology, University of Vienna, 1030 Vienna, Austria*

*^3^Solid-state NMR Application, Bruker Biospin GmbH, Rheinstetten, Germany*

**Correspondence should be sent to gil.goobes@biu.ac.il*


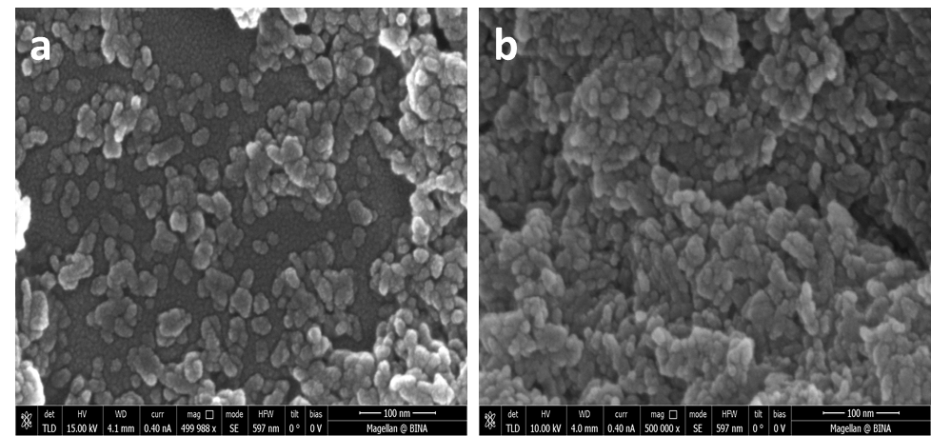


**Figure S1.** Platelet-shaped crystallites observed in the transmission electron micrographs of (a) HAP and (b) HAP•qOPN. Agglutination of apatite particles observed from scanning electron micrographs of (a) HAP and (b) HAP•qOPN.

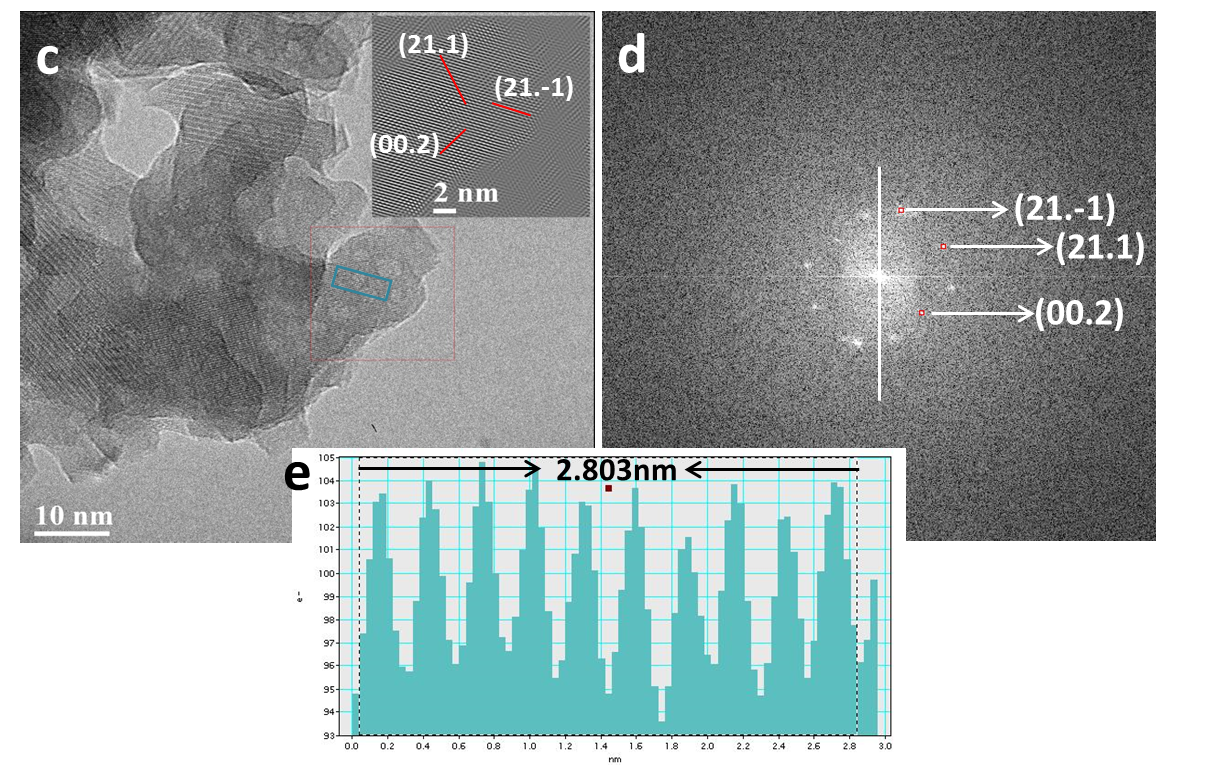


**Figure S2.** (**a**) A typical bright field image of the HAP•OPN sample. (**b**) Selected area electron diffraction (SAED) of the region in (a) marked with red square. The reflections are indexed in terms of hexagonal hydroxyapatite (S.G. P63/m (176)); d00.2 = 3.44Å and the third ring comprises the three reflections of the family of planes, d30.0 = 2.72Å, d11.2 = 2.78Å, and d21.1 = 2.81Å. (**c**) A bright field image from another region in HAP•OPN. **inset** in (**c**) displays the atomic resolution of HAP•OPN from the area marked by the red square marking the area from where the SAED was taken with a 300 nm aperture from the area presented in image. (**d**) Fast Fourier Transform (FFT) was taken from the area marked by the red square (image (**c**)); d00.2 = 3.44Å, d21.1 = 2.81Å, and d21.-1 = 2.81Å. (**e**) The intensity profile taken from the area marked by cyan rectangular. It shows measurements for 10 lattices.

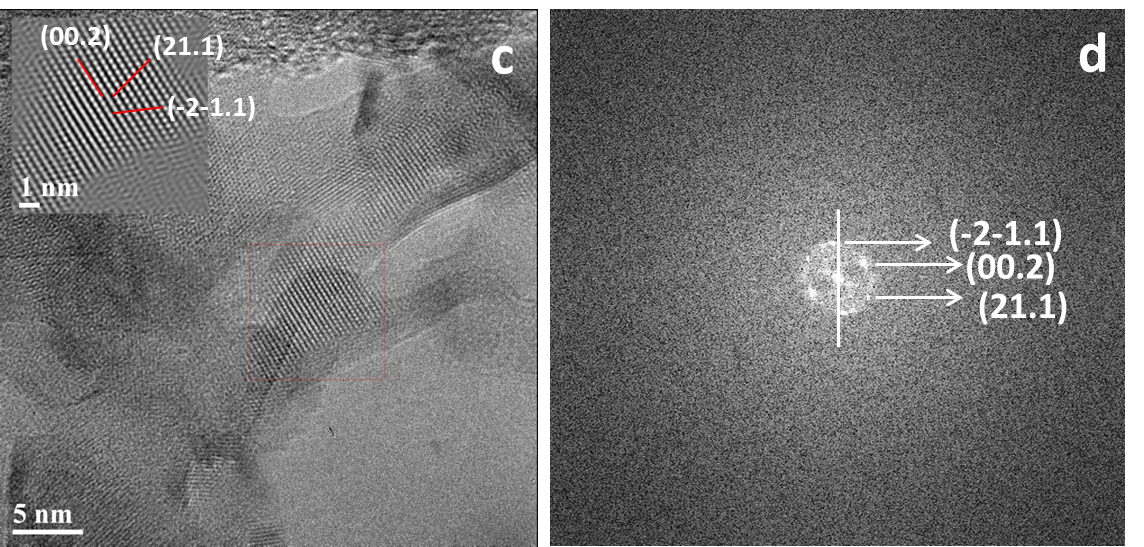


**Figure S3**. (**a**) A typical bright field image of the HAP sample. (**b**) SAED of the region in (a) marked with red square. The reflections are indexed in terms of the hexagonal hydroxyapatite (S.G. P63/m (176)) d10.0 = 8.22Å, d11.0 = 4.81Å, d20.0 = 4.11Å, d00.2 = 3.49Å, d21.0 = 3.08Å and d30.0 = 2.74Å. (**c**) Another typical bright field image of the HAP sample. Red square marks the area from where electron diffraction shown in **(d)** was taken with a 300 nm aperture. The **inset** in (**c**) displays the atomic resolution of HAP from the area marked by the red square. (**d**) Fast Fourier Transform (FFT) was taken from the area marked by the red square in (**c**); d00.2 = 3.41Å, d21.1 = 2.81Å, and d-2-1.1 = 2.81Å.


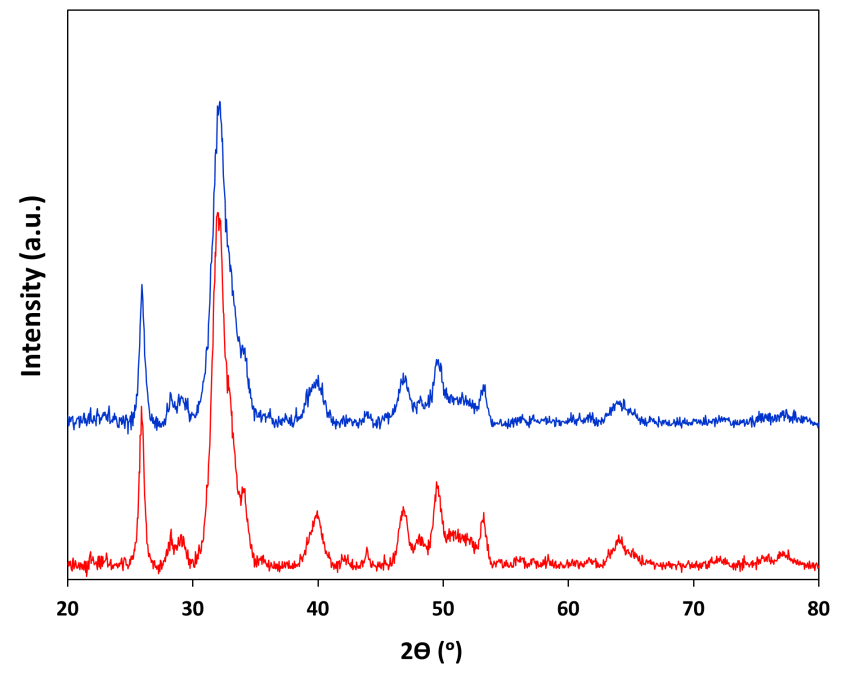
 **Figure S4.** Powder XRD patterns of non-annealed synthetic HAP (red) and HAP•qOPN (blue).


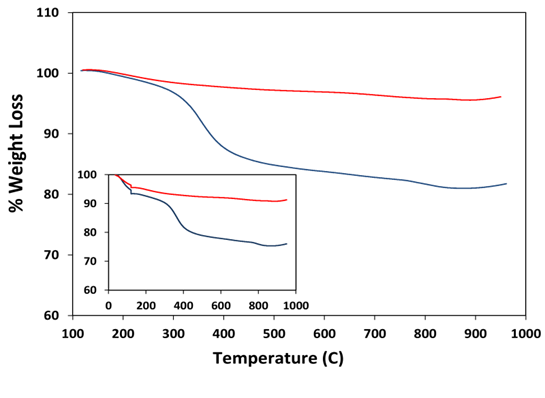


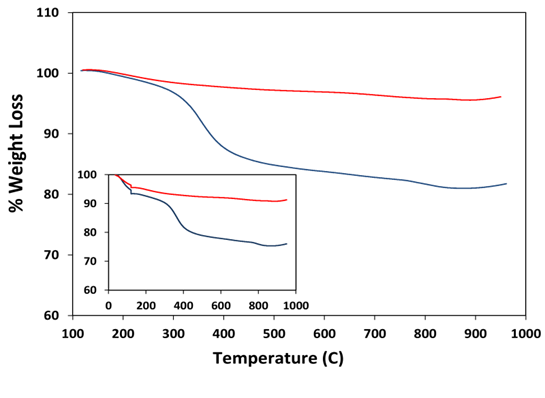

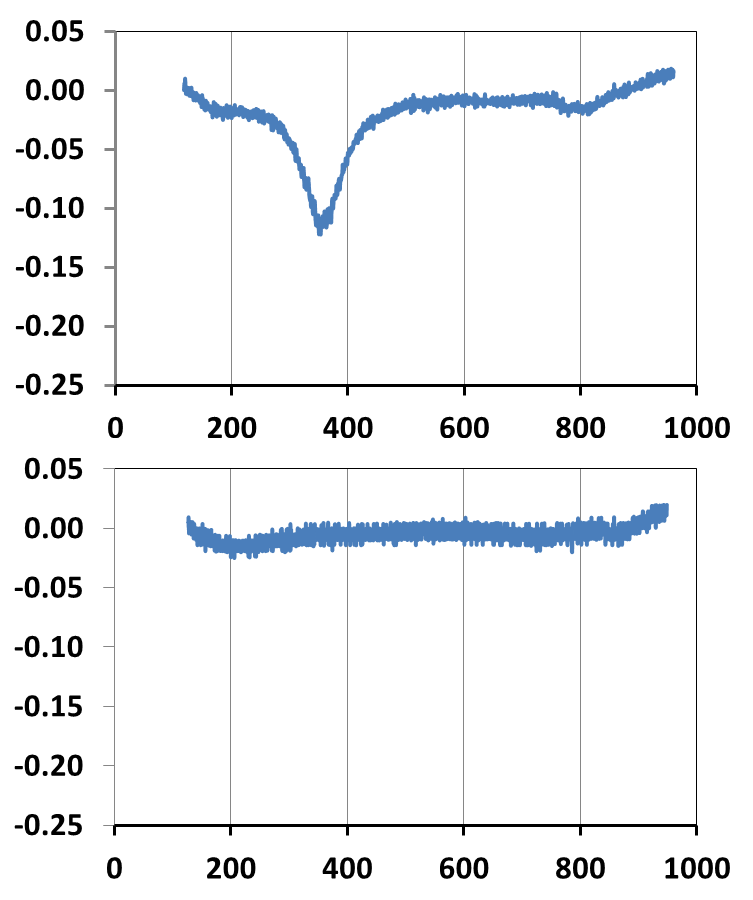


**Figure S5**. **(top)** Normalized TGA analysis of HAP (red) and HAP•qOPN (blue). **Inset**: Original TGA curves including weight of water lost by evaporation at temperatures below 120 ᵒC. **(middle and bottom)** Differential thermal analysis (DTA) of thermogravimetric analysis data shown in figure 4. **(middle)** DTA graph of HAP•OPN and **(bottom)** DTA graph of HAP. Graphs were obtained by numerically differentiating the TGA data using a 5-point Newton-Raphson method and then denoising using 5-point moving average repeated twice.


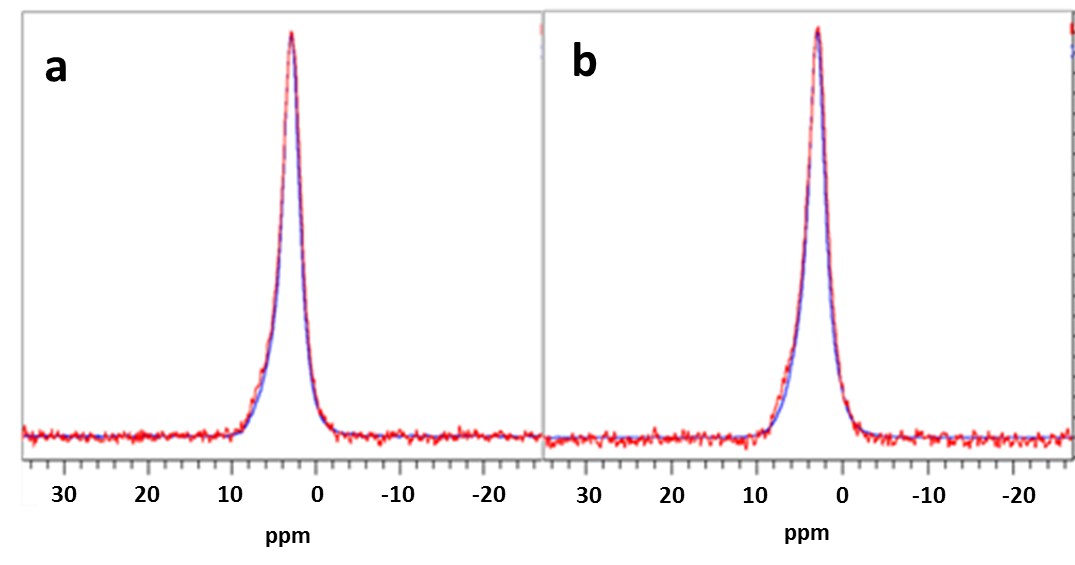


**^31^P frequency (ppm)**

**Figure S6.** **(a)** ^31^P direct polarization (DP) and **(b)** ^31^P cross polarization (CP) spectra of HAP (blue), and HAP•OPN (red) showing phosphate species in the two materials. CP spectra were measured using a contact time of 3 ms.


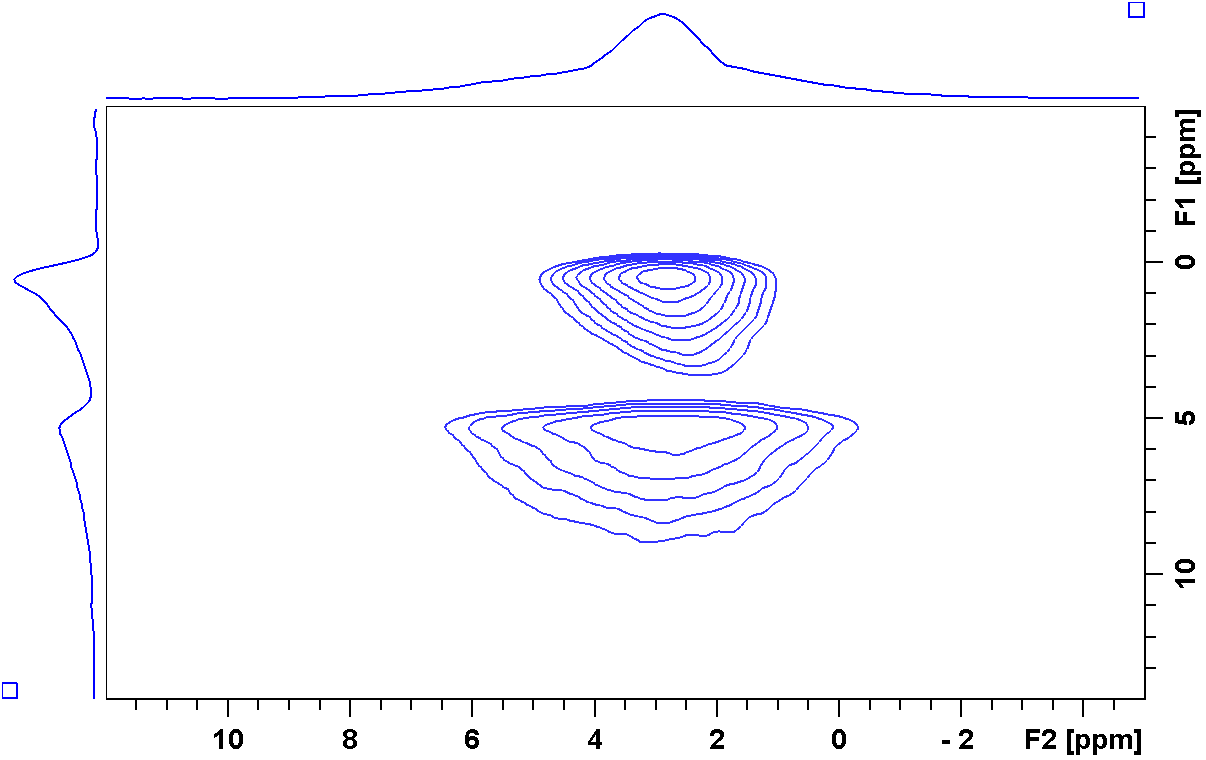

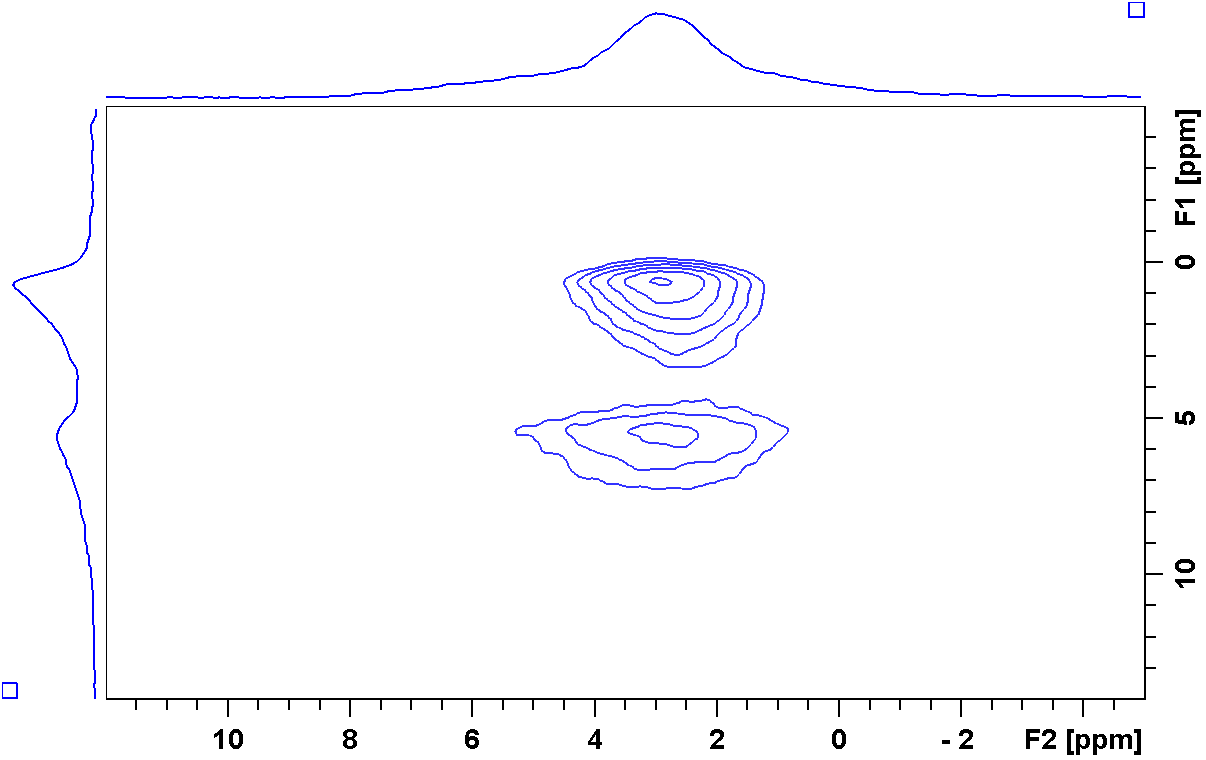


**Figure S7**. The same 2D ^1^H-^31^P HETCOR spectra of HAP (a) and HAP•qOPN (b) shown in Figure S3 with 1D skyline projections shown along the ^1^H axis (vertical) and ^31^P axis (horizontal).


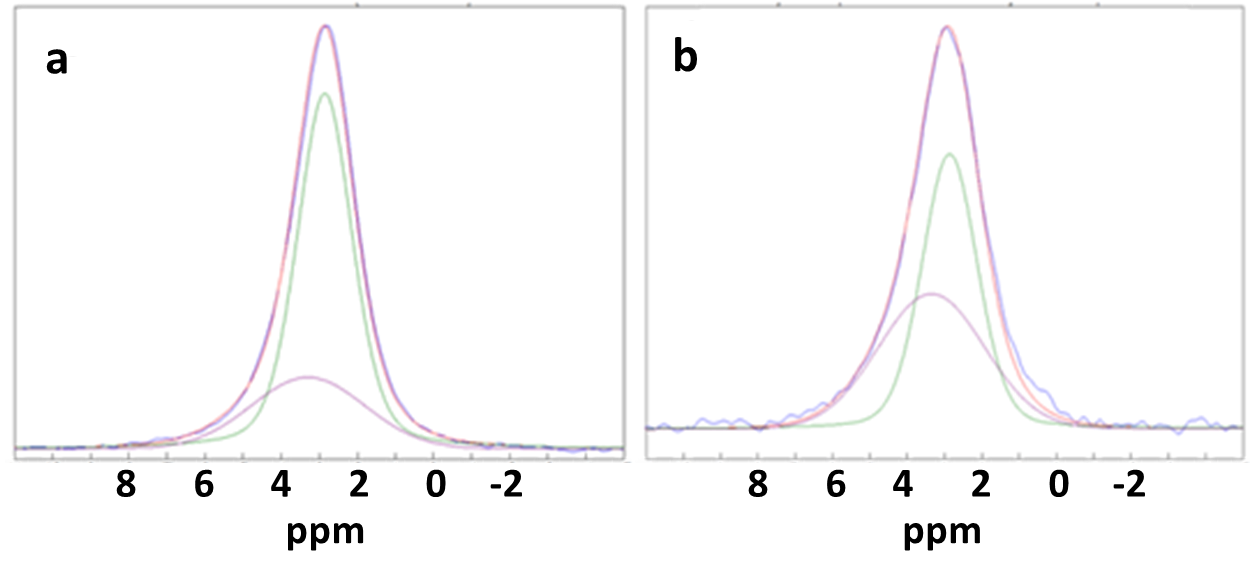


**^31^P frequency (ppm)**

**Figure S8**. ^31^P slice (blue) along the OH^-^ resonance in the 2D ^1^H-^31^P HETCOR spectra of (a) HAP and (b) HAP•OPN with two-line fitting (red). The individual phosphate resonances discerned from the deconvolution procedure are shown in green and in purple.


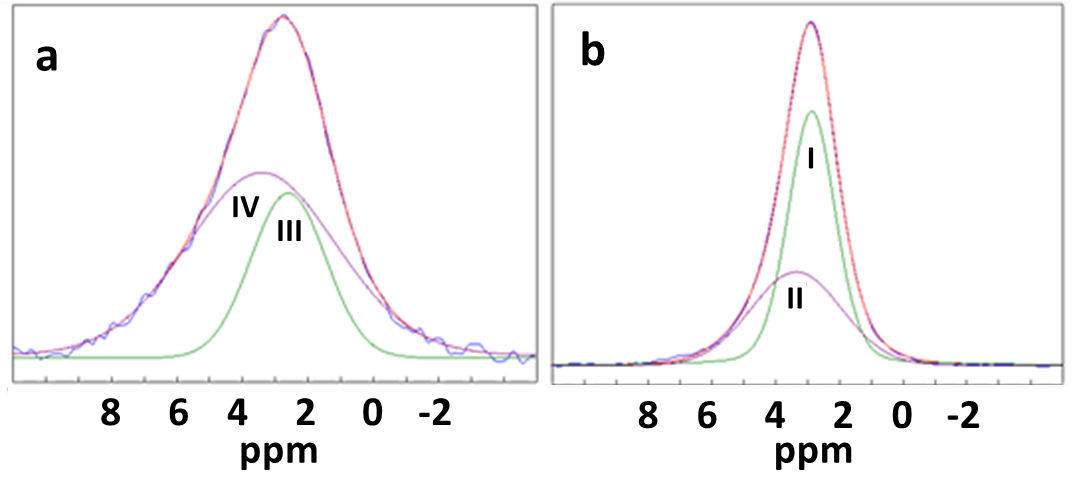


**^31^P frequency (ppm)**

**Figure S9**. ^31^P slices (blue) along the H_2_O **(a)** and along the OH^-^ **(b)** resonance in the 2D ^1^H-^31^P HETCOR spectrum of HAP•OPN with two-line fitting (red). Each line comprises two resonances (green and purple) giving a total of four phosphate resonances (I-IV) representing different phosphate species. Resonance I is associated with highly ordered phosphates in the apatite crystal, resonance IV is associated with highly disordered surface layer phosphates. The other two phosphates are spatially located in between these two mineral layers. Resonance III represents partially disordered phosphates excited by water protons while resonance II represents partially ordered phosphates excited by OH^-^ protons.

**Figure S10.** Pulse scheme of 1D z-filtered CHHP experiment. The phases of pulses are cycled according to: $\boldsymbol{\phi}_{\mathbf{1}}\mathbf{=}1_{16}3_{16}$ **,** $\boldsymbol{\phi}_{\mathbf{2}}=0$, $\boldsymbol{\phi}_{\mathbf{3}}=1$, $\boldsymbol{\phi}_{\mathbf{4}}=3 1$, $\boldsymbol{\phi}_{\mathbf{5}}=0$ , $\boldsymbol{\phi}_{\mathbf{6}}=$ 0 0 1 1 2 2 3 3 , $\boldsymbol{\phi}_{\mathbf{7}}=1 1 0 0 3 3 2 2 , \boldsymbol{\phi}_{\mathbf{8}}= 3 3 0 0 1 1 2 2 1 1 2 2 3 3 0 0$ , $\boldsymbol{\phi}_{\mathbf{9}}=0 0 1 1 2 2 3 3 ,$ $\boldsymbol{\phi}_{\mathbf{10}}=0 0 1 1 2 2 3 3$, $\boldsymbol{\phi}_{\boldsymbol{rec}}= 0 2 3 1 2 0 1 3 2 0 1 3 0 2 3 1 2 0 1 3 0 2 3 1 0 2 3 1 2 0 1 3$


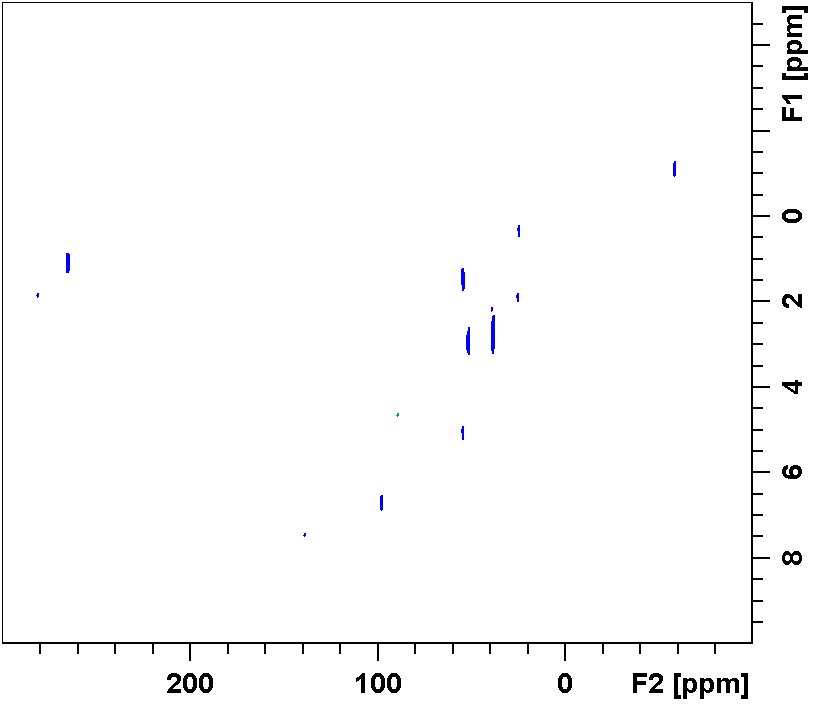


**^31^P frequency (ppm)**

**^13^C frequency (ppm)**

**Figure S11.** 2D ^31^P-^13^C double-CP spectrum of HAP•[U-^13^C 99%,^15^N 95%]qOPN (full spectrum) with contact time of 5 ms between ^1^H and ^31^P, and 16 ms contact time between ^31^P and ^13^C.


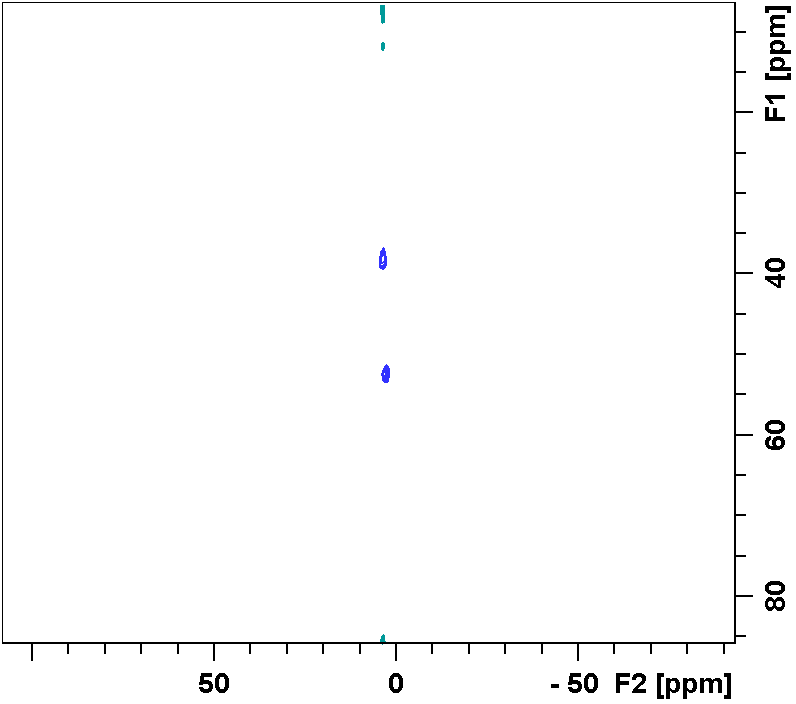


**^13^C frequency (ppm)**

**^31^P frequency (ppm)**

**Figure S12.** 2D ^31^P-^13^C rotor synchronized z-filtered TEDOR spectrum of HAP•[U-^13^C 99%,^15^N 95%]qOPN (full spectrum) for the aliphatic region at a spinning rate of 10 kHz.


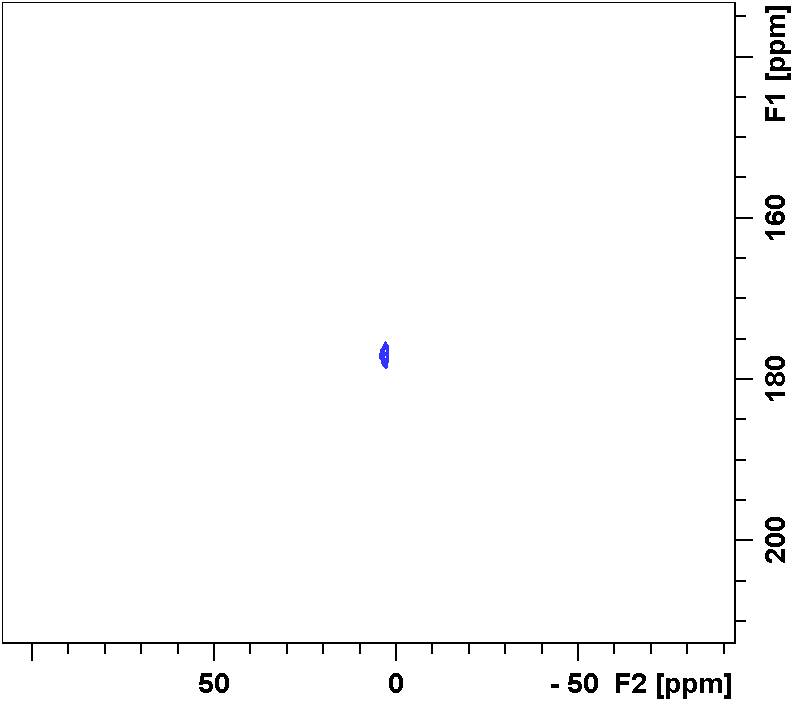


**^13^C frequency (ppm)**

**^31^P frequency (ppm)**

**Figure S13.** 2D ^31^P-^13^C rotor synchronized z-filtered TEDOR spectrum of HAP•[U-^13^C 99%,^15^N 95%]qOPN (full spectrum) for the carbonyl region at a spinning rate of 10 kHz.

**Table S1. Elemental analysis of HAP and HAP•qOPN**

|  | Nitrogen | Carbon | Hydrogen | Oxygen |
| --- | --- | --- | --- | --- |
| HAP | - | 1.13 | 1.39 | 9.71 |
| HAP•qOPN | 2.21 | 7.79 | 1.81 | 14.24 |

**Table S2. Deconvolution results of one-dimensional ^31^P projections of HAP and HAP•qOPN**


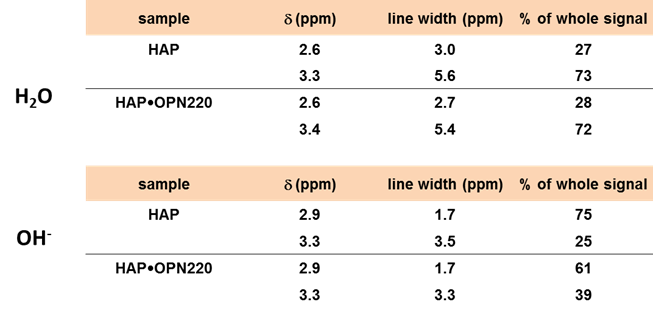


**HAP•qOPN**

**HAP•qOPN**

**HAP**

**HAP**
